# High sugar diet promotes tumor progression paradoxically through aberrant upregulation of *pepck1*

**DOI:** 10.1007/s00018-024-05438-2

**Published:** 2024-09-11

**Authors:** Che-Wei Chang, Yu-Hshun Chin, Meng-Syuan Liu, Yu-Chia Shen, Shian-Jang Yan

**Affiliations:** 1 Institute of Basic Medical Sciences, College of Medicine, National Cheng Kung University, No. 1, University Road, Tainan City, Taiwan; 2 Department of Physiology, College of Medicine, National Cheng Kung University, No. 1, University Road, Tainan City, Taiwan

**Keywords:** Hallmarks of cancer, Gene regulation, Cancer metabolism, Signal transduction, Model organisms, Epigenetics

## Abstract

High dietary sugar (HDS), a contemporary dietary concern due to excessive intake of added sugars and carbohydrates, escalates the risk of metabolic disorders and concomitant cancers. However, the molecular mechanisms underlying HDS-induced cancer progression are not completely understood. We found that phosphoenolpyruvate carboxykinase 1 (PEPCK1), a pivotal enzyme in gluconeogenesis, is paradoxically upregulated in tumors by HDS, but not by normal dietary sugar (NDS), during tumor progression. Targeted knockdown of *pepck1*, but not *pepck2*, specifically in tumor tissue in *Drosophila *in vivo, not only attenuates HDS-induced tumor growth but also significantly improves the survival of Ras/Src tumor-bearing animals fed HDS. Interestingly, HP1a-mediated heterochromatin interacts directly with the *pepck1* gene and downregulates *pepck1* gene expression in wild-type *Drosophila*. Mechanistically, we demonstrated that, under HDS conditions, *pepck1* knockdown reduces both wingless and TOR signaling, decreases evasion of apoptosis, reduces genome instability, and suppresses glucose uptake and trehalose levels in tumor cells in vivo. Moreover, rational pharmacological inhibition of PEPCK1, using hydrazinium sulfate, greatly improves the survival of tumor-bearing animals with *pepck1* knockdown under HDS. This study is the first to show that elevated levels of dietary sugar induce aberrant upregulation of PEPCK1, which promotes tumor progression through altered cell signaling, evasion of apoptosis, genome instability, and reprogramming of carbohydrate metabolism. These findings contribute to our understanding of the complex relationship between diet and cancer at the molecular, cellular, and organismal levels and reveal PEPCK1 as a potential target for the prevention and treatment of cancers associated with metabolic disorders.

**Supplementary Information:**

The online version contains supplementary material available at 10.1007/s00018-024-05438-2.

## Introduction

High dietary sugar (HDS) is a contemporary health issue characterized by elevated consumption of total sugars, including both added and free sugars, which escalates the risk of metabolic disorders and their associated cancers [1]. Indeed, diet is a key lifestyle factor that significantly contributes to increased cancer risk; however, the mechanisms remain unclear and await further investigation [2]. Therefore, investigating the role of HDS-induced metabolic dysfunction in promoting cancer progression is essential for the prevention and treatment of diet-associated cancers. Across diverse animal models, HDS has been observed to enhance tumor growth and promote cancer progression through modifications in growth signaling and metabolic states within cancer cells [3–5]. Under HDS conditions, cancer cells exhibit the capacity to adapt their growth signaling and metabolic states, potentially involving upregulation of the wingless/Wnt pathway and mTOR signaling, along with evasion of apoptosis and genome instability, which contributes to elevated mutation rates [6–9]. However, the precise molecular mechanisms underlying HDS-induced cancer progression via metabolic reprogramming remain unclear.

Insufficient provision of glucose often works against the increased nutritional requirements of tumors, highlighting the importance of gluconeogenesis, a critical glucose anabolism process for synthesis of glucose from noncarbohydrate precursors, in fueling tumor cell growth [10, 11]. Phosphoenolpyruvate carboxykinases (PEPCKs or PCKs), including PEPCK1 (cytosolic isoform) and PEPCK2 (mitochondrial isoform), catalyze the first rate-limiting step in the gluconeogenesis pathway and exhibit conserved functions across species [12]. Research has underscored the role of PEPCKs in promoting cancer, particularly PEPCK1, which facilitates colon cancer growth by increasing gluconeogenesis metabolites through phosphoenolpyruvate and pyruvate production, along with mTOR signaling activation [10, 13, 14]. Additionally, PEPCK1 inhibition has been shown to decrease colon cancer cell growth by downregulating lactate utilization [15]. However, whether and how PEPCK1/2 regulate HDS-induced cancer progression remain completely unexplored.

In this study, we utilized *Drosophila* Ras/Src cancer models to explore the role of paradoxical *pepck1*/2 upregulation in HDS-induced tumor progression. Taking advantage of the genetic conservation between *Drosophila melanogaster* and humans, particularly in genes associated with disease, such as oncogenes and tumor suppressor genes, we investigated the impact of *pepck1/2* upregulation in Ras/Src-induced tumor growth [16]. Notably, evolutionary conservation of the Ras protein family underscores its pivotal role in regulating cellular growth, differentiation, and survival signaling, while C-terminal Src kinase (csk) functions as a crucial regulatory protein primarily involved in the negative regulation of Src family proteins [17].

Our prior studies have uncovered new epigenetic functions of heterochromatin formation, mediated by heterochromatin protein 1a (HP1a), in inhibiting HDS-induced tumor progression [18]. However, the mechanisms by which HDS-induced tumor progression occurs, particularly through metabolic reprogramming, are not fully understood. Here, through an unbiased bioinformatics approach, we surprisingly identified *pepck1* as a gene significantly upregulated in tumors when exposed to HDS, whereas such an upregulation does not occur with normal dietary sugar (NDS). We also demonstrated that PEPCK1 is a direct target of regulation via HP1a-mediated heterochromatin formation. Interestingly, we also found that *pepck2* was upregulated, though to a lesser extent than *pepck1*, during HDS-induced tumor progression. To elucidate the role of these pivotal gluconeogenesis genes in HDS-induced tumor growth, we created *ras*^*G12V*^*, pepck1*^*RNAi*^*; csk*^*−/−*^ and *ras*^*G12V*^*, pepck2*^*RNAi*^*; csk*^*−/−*^* Drosophila* models that carry Ras/Src-induced tumors with *pepck1* and *pepck2* RNAi knockdown, respectively. This approach effectively decreased the excessive *pepck1* or *pepck2* expression observed in *ras*^*G12V*^*; csk*^*−/−*^ tumor-bearing animals. Moreover, we also created *Drosophila* models that carry Ras/Src-induced tumors with *pepck1* or *pepck2* overexpression, respectively. Importantly, knockdown of *pepck1*, but not *pepck2*, within tumor cells not only diminishes the growth induced by HDS but also markedly enhances the survival rate of animals bearing Ras/Src tumors when they are fed HDS. Interestingly, overexpression of *pepck1* or *pepck2* did not further exacerbate tumor growth/developmental delay/lethality in animals bearing Ras/Src tumors under HDS. Furthermore, knockdown of *pepck1* diminishes wingless and TOR signaling, decreases apoptosis and genome instability, and suppresses HDS-induced upregulation of glucose uptake and trehalose levels in tumor cells. Moreover, pharmacological inhibition of PEPCK1 using hydrazinium sulfate (HS) significantly enhances the survival of animals carrying Ras/Src tumors with knocked down expression of *pepck1* under both NDS and HDS conditions.

Thus, our study reveals novel mechanisms by which aberrant upregulation of *pepck1* promotes HDS-induced tumor progression, including enhanced wingless/Wnt and mTOR/TOR signaling, apoptosis evasion, genome instability, and metabolic reprogramming with increased glucose uptake and trehalose levels. These findings provide greater understanding of the intricate relationship between diet and cancer and reveal potential targets for the prevention and treatment of cancers associated with metabolic disorders.

## Results

### *pepck1* is upregulated during HDS-induced tumor progression

Our prior research has shown that HDS decreases heterochromatin levels in tumor cells, and HP1a-mediated heterochromatin formation suppresses the tumor progression induced by HDS [18]. These effects have raised questions about the specific downstream genes involved in the process of HDS-induced tumor progression through HP1a-mediated heterochromatin formation. By analyzing microarray databases from cultured *Drosophila* cells treated with HP1a RNAi and from *Drosophila* fed an HDS regimen [19–21], we identified several metabolic genes, including *phosphoenolpyruvate carboxykinases1* (*pepck1*), as potential targets regulated by both HP1a and HDS (Supplementary Fig. S1A). To further explore how HP1a-mediated heterochromatin formation regulates metabolism, we utilized an RU486-inducible system for HP1a overexpression or knockdown in normal adult flies. Consistently, analysis revealed that levels of *pepck1* were elevated in flies with HP1a knockdown and reduced in those with HP1a overexpression (Supplementary Fig. S1B, C). This suggests that HP1a-mediated heterochromatin formation plays a role in the downregulation of *pepck1* in carbohydrate metabolism. This observation was further supported by chromatin immunoprecipitation (ChIP) assays using an H3K9me2 antibody, which demonstrated that heterochromatin is present in the *pepck1* gene body (Supplementary Fig. S1D). Collectively, these findings suggest that heterochromatin formed through an HP1a-mediated process interacts directly with the *pepck1* gene to downregulate *pepck1* expression in vivo.

We then undertook a comprehensive investigation of transcriptional changes in glycolysis or gluconeogenesis genes following HDS-induced tumor progression. Experiments using tumor tissues from HDS-fed third instar larvae revealed increased expression of both cytosolic (*pepck1*) and mitochondrial (*pepck2*) forms of *pepck* mRNA, as well as elevated *lactate dehydrogenase* (*Ldh*) mRNA, indicative of the Warburg Effect in HDS-induced tumors (Supplementary Fig. S2A–E).

**Fig. 1:**
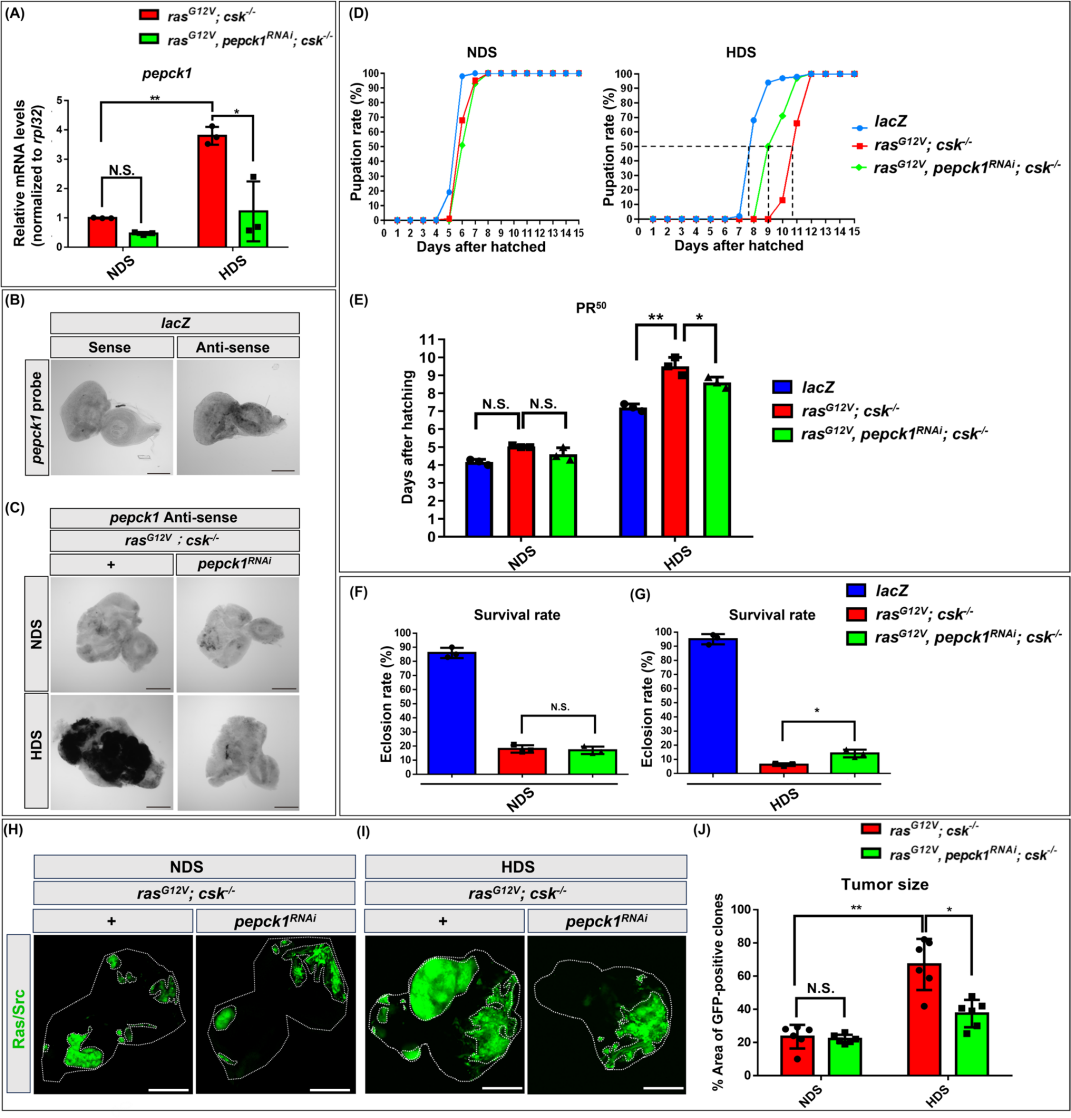
Reducing HDS-induced upregulation of *pepck1* alleviates developmental delay and reduces lethality and tumor size among Ras/Src tumor-bearing animals. *Drosophila* with the following genotypes were used in these experiments: *lacZ* (control), *ras*^*G12V*^*; csk*^*−/−*^ (tumor-bearing), and *ras*^*G12V*^*, pepck1*^*RNAi*^*; csk*^*−/−*^ (tumor-bearing with *pepck1* knockdown). **A** Relative *pepck1* mRNA levels were determined based on RNA extracted from 30 eye discs of combined 3rd instar male and female larvae (n = 15) fed HDS. Results were normalized to *rpl32.* **B** *pepck1* mRNA expression patterns were examined using DIG-labeled *pepck1* sense (negative control) or antisense RNA probes in WT male eye discs. **C** *pepck1* mRNA expression patterns were analyzed using DIG-labeled *pepck1* antisense RNA probes in the eye discs of male *ras*^*G12V*^*; csk*^*−/−*^ and *ras*^*G12V*^*, pepck1*^*RNAi*^*; csk*^*−/−*^* Drosophila* under NDS or HDS. **D** Pupation rates of combined male and female animals fed a 0.15 M sucrose diet (NDS) and a 0.75 M sucrose diet (HDS). **E** Number of days (PR^50^) until pupation rate reached 50% among control and tumor-bearing animals. **F**, **G** Eclosion rates of combined male and female animals fed NDS or HDS. (H) Eye discs from 3rd instar *ras*^*G12V*^*; csk*^*−/−*^ and *ras*^*G12V*^*, pepck1*^*RNAi*^*; csk*^*−/−*^ female *Drosophila* larvae fed NDS, with GFP-labeled tumor cells. **I** Eye discs from 3rd instar *ras*^*G12V*^*; csk*^*−/−*^ and *ras*^*G12V*^*, pepck1*^*RNAi*^*; csk*^*−/−*^ female *Drosophila* larvae fed HDS, with GFP-labeled tumor cells. **J** Percentage of GFP-positive tumor cells normalized to total eye disc area from female *Drosophila* fed NDS or HDS. Results are shown as mean ± SD. Asterisks indicate statistically significant differences via two-way ANOVA with paired controls (*P < 0.05, **P < 0.01). *GFP* green fluorescent protein, *HDS* high dietary sugar, *NDS* normal dietary sugar, *N.S.* not significant; *SD* standard deviation

To investigate the role of PEPCK1 in HDS-induced tumor progression, we generated a *pepck1* RNAi strain in Ras/Src tumor-bearing animals to specifically reduce *pepck1* expression in tumors under HDS (Fig. 1). First, we assessed the efficacy of the transgenic *pepck1 RNAi* in flies, noted as *pepck1*^*RNAi*^, and found a significant decrease in *pepck1* mRNA levels in the Ras/Src tumor-bearing strain when it was combined with the *pepck1 RNAi* line (Fig. 1A; Supplementary Fig. S2B). Subsequently, we investigated *pepck1* expression patterns in tumors using RNA in situ hybridization with digoxygenin (DIG)-labeled pepck1 antisense RNA probes in the eye discs of male *ras*^*G12V*^*; csk*^*−/−*^ and *ras*^*G12V*^*, pepck1*^*RNAi*^*; csk*^*−/−*^ tumor-bearing animals under NDS or HDS conditions. The results revealed consistently increased *pepck1* mRNA expression in HDS-induced tumor cells and validated efficient knockdown of *pepck1* by RNAi in vivo (Fig. 1B, C).

Developmental delay and lethality are increased in tumor-bearing animals exposed to HDS [18]. Therefore, we investigated whether reducing *pepck1* expression could reverse these defects in *ras*^*G12V*^*, pepck1 *^*RNAi*^*; csk*^*−/−*^ tumor-bearing animals. Remarkably, *pepck1* knockdown was associated with significant reduction in developmental delay and increased survival rates of animals compared to the *ras*^*G12V*^*; csk*^*−/−*^ strain under HDS conditions (Fig. 1D–G). Next, we investigated whether the knockdown of *pepck1* in tumors could alleviate tumor burden. Indeed, the tumor-bearing animals with *pepck1* knockdown (*ras*^*G12V*^*, pepck1*^*RNAi*^*; csk*^*−/−*^) showed notable reduction in GFP-positive tumor growth compared to tumor-bearing animals without *pepck1* knockdown (*ras*^*G12V*^*; csk*^*−/−*^) (Fig. 1H–J). This finding suggests that PEPCK1 plays an important role in promoting the progression of HDS-induced tumors.

Moreover, consistent with qPCR results (Fig. S2A–D), RNA in situ hybridization with an antisense *pepck2* probe showed that *pepck2* was upregulated during HDS-induced tumor progression, though to a lesser extent than *pepck1* (Fig. S3A–C). However, the knockdown of *pepck2* in tumor cells did not lead to significant changes in tumor growth, developmental delay, or lethality in Ras/Src tumor-bearing animals under HDS (Fig. S3D–H). Likewise, overexpression of *pepck2* had no effect on tumor growth, developmental delay, or lethality in animals bearing Ras/Src tumors under HDS (Fig. S4A–F). Surprisingly, overexpression of *pepck1* marginally decreased the developmental delay, (Fig. S5A–C) but did not exacerbate tumor growth, induced by HDS in animals (Fig. S5D, E), nor did it alter the lethality of Ras/Src tumor-bearing animals fed HDS (Fig. S5F). Together, these data indicate that aberrant expression of *pepck1* in HDS-induced tumors contributes to developmental delay and reduced survival of animals, and knockdown of *pepck1*, but not *pepck2*, mitigates these effects, highlighting the essential role of *pepck1* in HDS-induced tumorigenesis.

### Knockdown of *pepck1* reduces tumor burden and wingless signaling in HDS-induced tumors

**Fig. 2:**
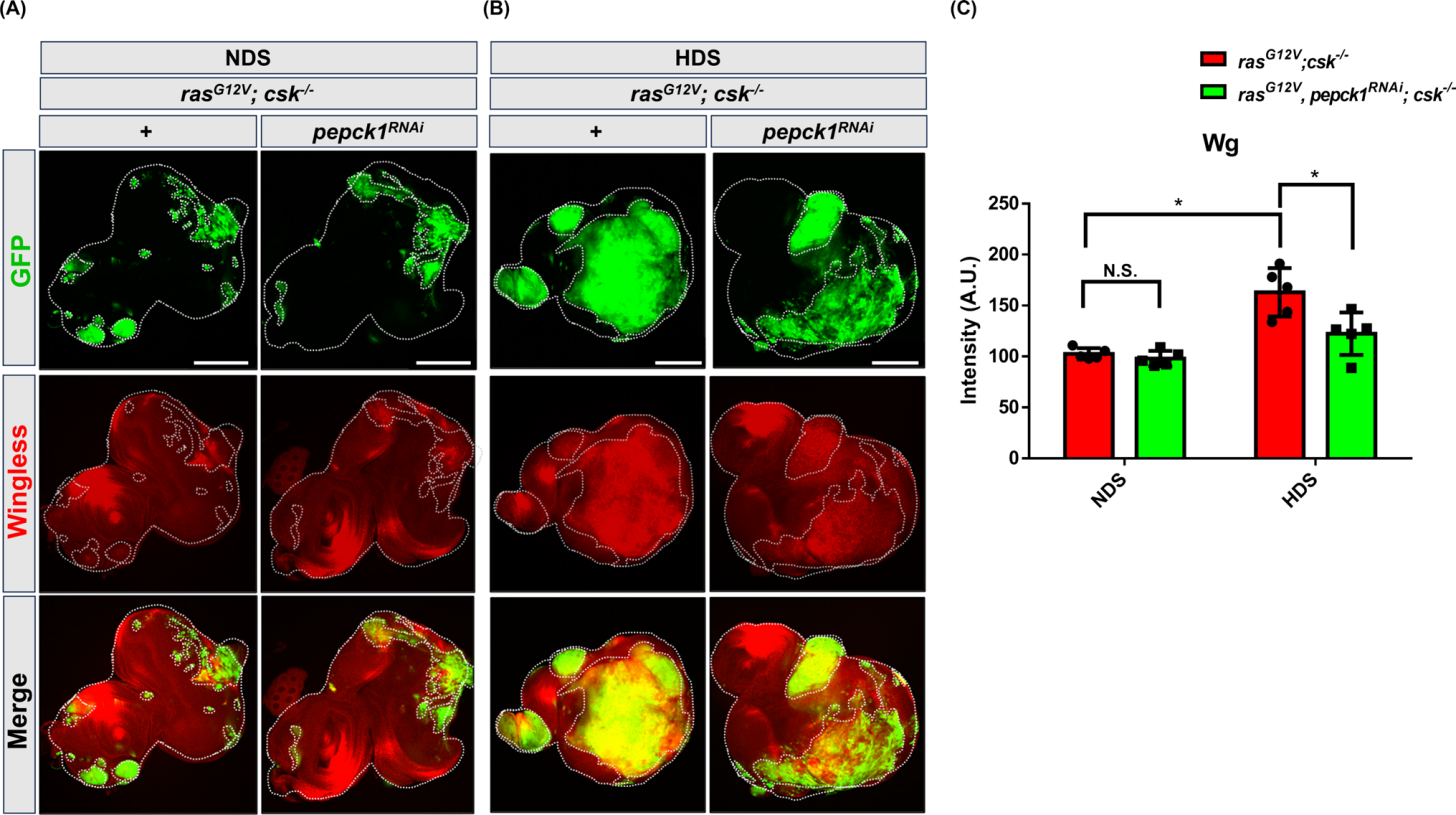
Knockdown of *pepck1* inhibits the expression of HDS-induced wingless in tumor cells. Eye discs from 3rd instar *ras*^*G12V*^*; csk*^*−/−*^ and *ras*^*G12V*^*, pepck1*^*RNAi*^*; csk*^*−/−*^* Drosophila* larvae, fed NDS or HDS, with GFP-labeled tumor cells and wingless immunostaining (red); Scale bar: 100 µm; n = 9 eye discs per group. **A** *ras*^*G12V*^*; csk*^*−/−*^ and *ras*^*G12V*^*, pepck1*^*RNAi*^*; csk*^*−/−*^ female *Drosophila* larvae fed NDS. **B** *ras*^*G12V*^*; csk*^*−/−*^ and *ras*^*G12V*^*, pepck1*^*RNAi*^*; csk*^*−/−*^ female *Drosophila* larvae fed HDS. **C** Quantification of wingless expression in tumor cells from female *Drosophila* fed NDS or HDS. n = 9 eye discs per group. Fluorescence intensity was quantified using the ZEISS ZEN Blue software. Results are shown as mean ± SD. Asterisk indicates a statistically significant difference via two-way ANOVA with paired control (*P < 0.05). *GFP* green fluorescent protein, *HDS* high dietary sugar, *NDS* normal dietary sugar, *SD* standard deviation

The wingless/Wnt pathway, a highly conserved regulatory pathway, governs both normal cell development and tumor cell proliferation and progression [22–25]. It has been observed that high dietary sugar (HDS) increases tumor cell insulin sensitivity and sugar influx, thereby promoting tumor progression through upregulation of wingless/Wnt signaling [6, 18, 26]. To assess whether *pepck1* knockdown reduces wingless/Wnt signaling in HDS-induced tumor growth, we utilized a specific antibody against wingless to evaluate wingless/Wnt signaling in tumor-bearing animals (*ras*^*G12V*^*; csk*^*−/−*^) and tumor-bearing animals with *pepck1* knockdown (*ras*^*G12V*^*, pepck1*^*RNAi*^*; csk*^*−/−*^) fed HDS or NDS. Compared to those without *pepck1* knockdown, tumor-bearing animals with *pepck1* knockdown exhibited a significant decrease in wingless protein expression within the tumor area during HDS-induced tumor growth (Fig. 2A–C). These results suggest that *pepck1* knockdown reduces wingless signaling during HDS-induced tumor progression. Overall, our findings suggest that PEPCK1 contributes to HDS-induced tumor growth by upregulating wingless signaling.

### Knockdown of *pepck1* reduces trehalose levels and glucose uptake in HDS-induced tumor cells

**Fig. 3:**
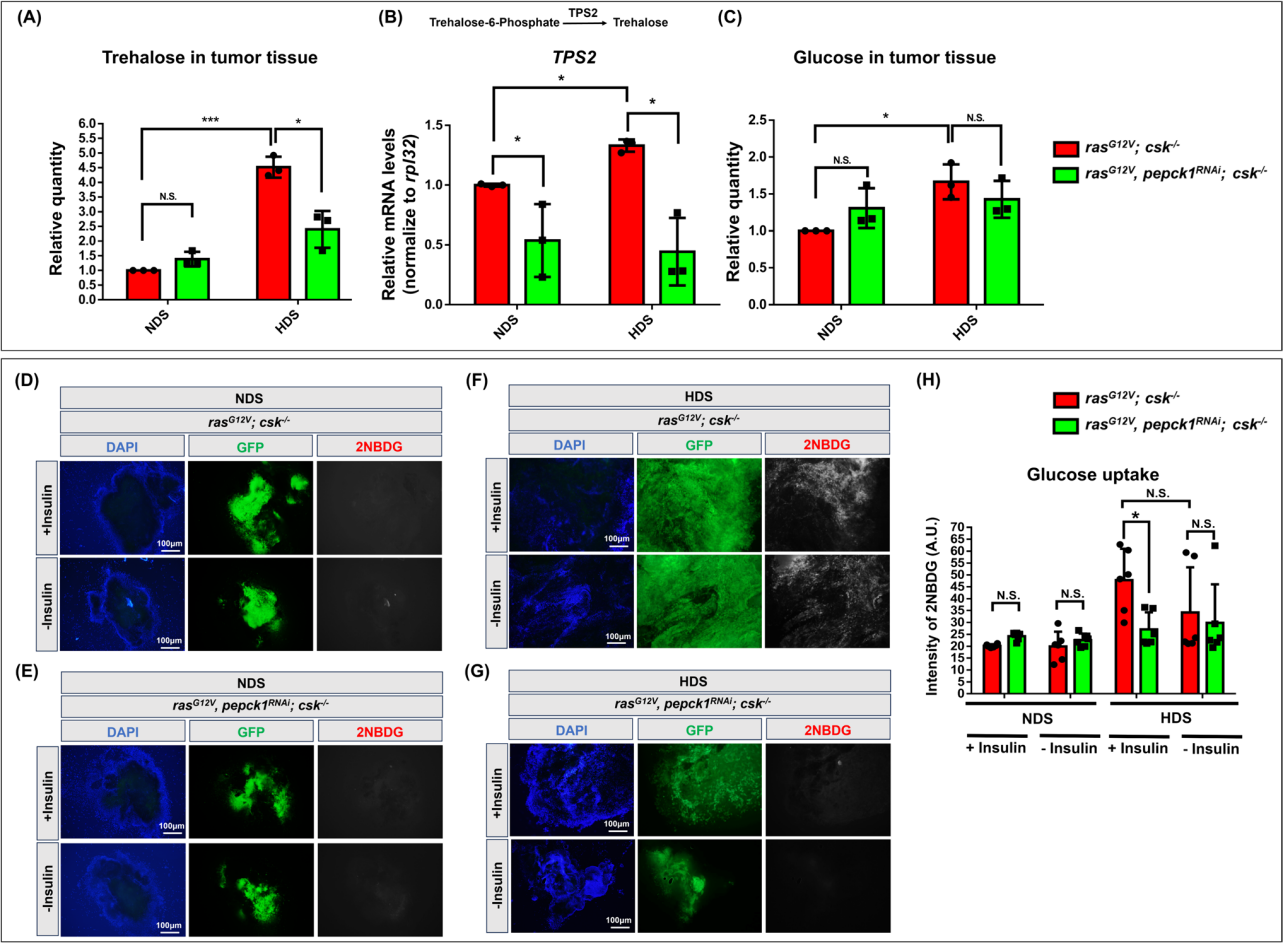
Knockdown of *pepck1* reduces HDS-induced elevated trehalose levels and glucose uptake in tumor cells. **A** Trehalose levels were measured using 60 eye discs from combined 3rd instar *ras*^*G12V*^*; csk*^*−/−*^ and *ras*^*G12V*^*, pepck1*^*RNAi*^*; csk*^*−/−*^ male and female *Drosophila* larvae fed NDS or HDS. **B** Relative levels of *TPS2 (trehalose-6-phosphate phosphatase)* mRNA were determined based on RNA extracted from 30 eye discs of combined 3rd instar *ras*^*G12V*^*; csk*^*−/−*^ and *ras*^*G12V*^*, pepck1*^*RNAi*^*; csk*^*−/−*^ male and female *Drosophila* larvae fed NDS or HDS. **C** Glucose levels were assessed using 60 eye discs from combined 3rd instar *ras*^*G12V*^*; csk*^*−/−*^ and *ras*^*G12V*^*, pepck1*^*RNAi*^*; csk*^*−/−*^ male and female *Drosophila* larvae fed NDS or HDS. Results are shown as mean ± SD. Asterisk indicates a statistically significant difference via two-way ANOVA with paired control (*P < 0.05; ***P < 0.001). **D**, **E** 2NBDG expression (red) in eye discs from 3rd instar *ras*^*G12V*^*; csk*^*−/−*^ and *ras*^*G12V*^*, pepck1*^*RNAi*^*; csk*^*−/−*^ female *Drosophila* larvae fed NDS, with GFP-labeled tumor cells (green). **F**, **G** Expression of 2NBDG (red) in eye discs from 3rd instar *ras*^*G12V*^*; csk*^*−/−*^ and *ras*^*G12V*^*, pepck1*^*RNAi*^*; csk*^*−/−*^ female *Drosophila* larvae fed HDS, with GFP-labeled tumor cells (green). **H** Quantification of 2NBDG levels (red) via fluorescence intensity in tumor cells (green) in eye discs from 3rd instar *ras*^*G12V*^*; csk*^*−/−*^ and *ras*^*G12V*^*, pepck1*^*RNAi*^*; csk*^*−/−*^ female *Drosophila* larvae fed NDS or HDS. Fluorescence intensity was quantified using the ZEISS ZEN Blue software. Results are shown as mean ± SD. Asterisks indicate statistically significant differences via two-way ANOVA with paired control (*P < 0.05). *GFP* green fluorescent protein, *HDS* high dietary sugar, *NDS* normal dietary sugar, *N.S.* not significant, *SD* standard deviation

In the adult *Drosophila* intestinal tumor model, tumor-bearing animals exhibit high levels of trehalose, which is a predominant sugar in the hemolymph [27–30]. Moreover, there is an increase in glucose uptake observed in Ras/Src tumor cells under HDS conditions [6]. Therefore, we next determined whether *pepck1* regulates trehalose and glucose levels during HDS-induced tumor progression. We observed elevated levels of trehalose and glucose in the HDS-induced tumor group and showed that reduction of *pepck1* in the tumor reversed the HDS-mediated increase in trehalose levels without affecting glucose levels (Fig. 3A, C). Moreover, *trehalose-6-phosphate phosphatase* (*T6Pase, TPS2*) mRNA, which encodes an enzyme that converts trehalose-6-phosphate into trehalose, was upregulated in HDS-induced tumors; importantly, knockdown of *pepck1* in Ras/Src tumors under both NDS and HDS conditions resulted in significant reduction of *T6Pase* levels (Fig. 3B). These results suggest that *pepck1* not only maintain trehalose homeostasis under NDS but also mediates HDS-induced elevation of trehalose levels in tumor cells through *T6Pase*. To further investigate whether *pepck1* regulates glucose uptake, we measured uptake rates of Ras/Src tumor tissue exposed to the glucose analog 2NBDG for a controlled period of time. HDS-induced tumors exhibited consistently elevated glucose uptake, and notably, reduction of *pepck1* levels decreased glucose uptake (Fig. 3D–H). Overall, these results suggest that the upregulation of PEPCK1 by HDS increases trehalose levels and glucose uptake, thereby enhancing glucose metabolism in tumor cells.

### Knockdown of *pepck1* reduces mTOR/TOR signaling during HDS-induced tumor progression

**Fig. 4:**
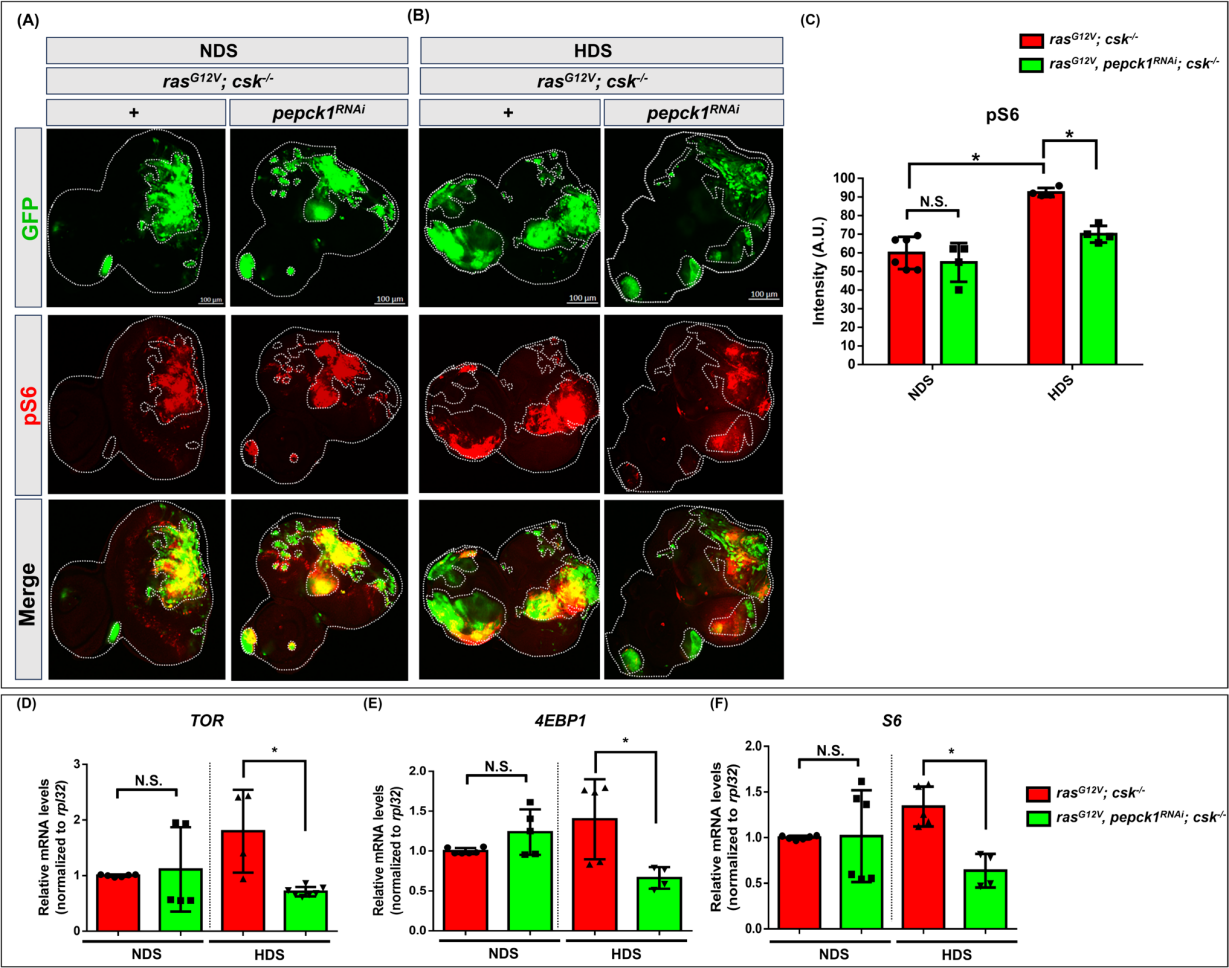
Knockdown of *pepck1* decreases TOR signaling in tumor cells under NDS and HDS. Eye discs from *ras*^*G12V*^*; csk*^*−/−*^ and *ras*^*G12V*^*, pepck1*^*RNAi*^*; csk*^*−/−*^ 3rd instar larvae, fed NDS or HDS, with GFP-labeled tumor cells (green) and pS6 immunostaining (red). Scale bar: 100 µm. **A** *ras*^*G12V*^*; csk*^*−/−*^ and *ras*^*G12V*^*, pepck1*^*RNAi*^*; csk*^*−/−*^ female larvae fed NDS. **B** *ras*^*G12V*^*; csk*^*−/−*^ and *ras*^*G12V*^*, pepck1*^*RNAi*^*; csk*^*−/−*^ female larvae fed HDS on day 7 AEL. **C** Quantification of pS6 fluorescence intensity in tumor cells from female larvae fed HDS; n = 9 eye discs per group. Fluorescence intensity was quantified using the ZEISS ZEN Blue software. Results are shown as mean ± SD of individual eye discs. Differences between groups were assessed via two-way ANOVA; *P < 0.05. Relative levels of *TOR* (**D**), *4EBP1* (**E**) and *S6* (**F**) mRNA were determined based on RNA extracted from 50 eye discs from combined 3rd instar *ras*^*G12V*^*; csk*^*−/−*^ and *ras*^*G12V*^*, pepck1*^*RNAi*^*; csk*^*−/−*^ male and female *Drosophila* larvae fed NDS or HDS. Results are shown as mean ± SEM. Asterisks indicate statistically significant differences via Student’s t-test (*P < 0.05). *AEL* after egg laying, *GFP* green fluorescent protein, *HDS* high dietary sugar, *NDS* normal dietary sugar, *SD* standard deviation

mTOR/TOR signaling, a crucial nutrient-sensing pathway, is upregulated in the progression of multiple types of cancer [31–35]. Previous work specifically demonstrated that TOR-S6K signaling is activated in Ras/Src tumors under a 1.0 M sucrose HDS diet, as assessed by phosho RpS6 (pS6) antibody staining [5]. To determine whether upregulated PEPCK1 enhances mTOR/TOR signaling and thus promotes tumor growth under HDS conditions, we assessed mTOR/TOR signaling activity using pS6 antibody, a specific antibody against phosphorylated Ribosomal protein S6 (RpS6) [36], in tumor cells. We observed reduction in pS6 staining intensity in the tumor areas in animals with *pepck1* knockdown after day 8 of HDS-induced tumor growth. Under NDS conditions, pS6 staining intensity in tumor tissue from animals with *pepck1* knockdown showed no significant difference compared to tissue from control tumor-bearing animals (Fig. 4A–C). We found that *pepck1* knockdown consistently resulted in significant reduction in the expression of *TOR*, *4EBP1,* and *S6* genes in tumor-bearing animals under HDS conditions, but not under NDS conditions (Fig. 4D–F). Therefore, *pepck1* knockdown suppressed TOR signaling associated with progression of HDS-induced tumors. These findings collectively suggest that PEPCK1 promotes mTOR/TOR signaling during HDS-induced tumor progression.

### Knockdown of *pepck1* reduces HDS-induced evasion of apoptosis in tumor cells

**Fig. 5:**
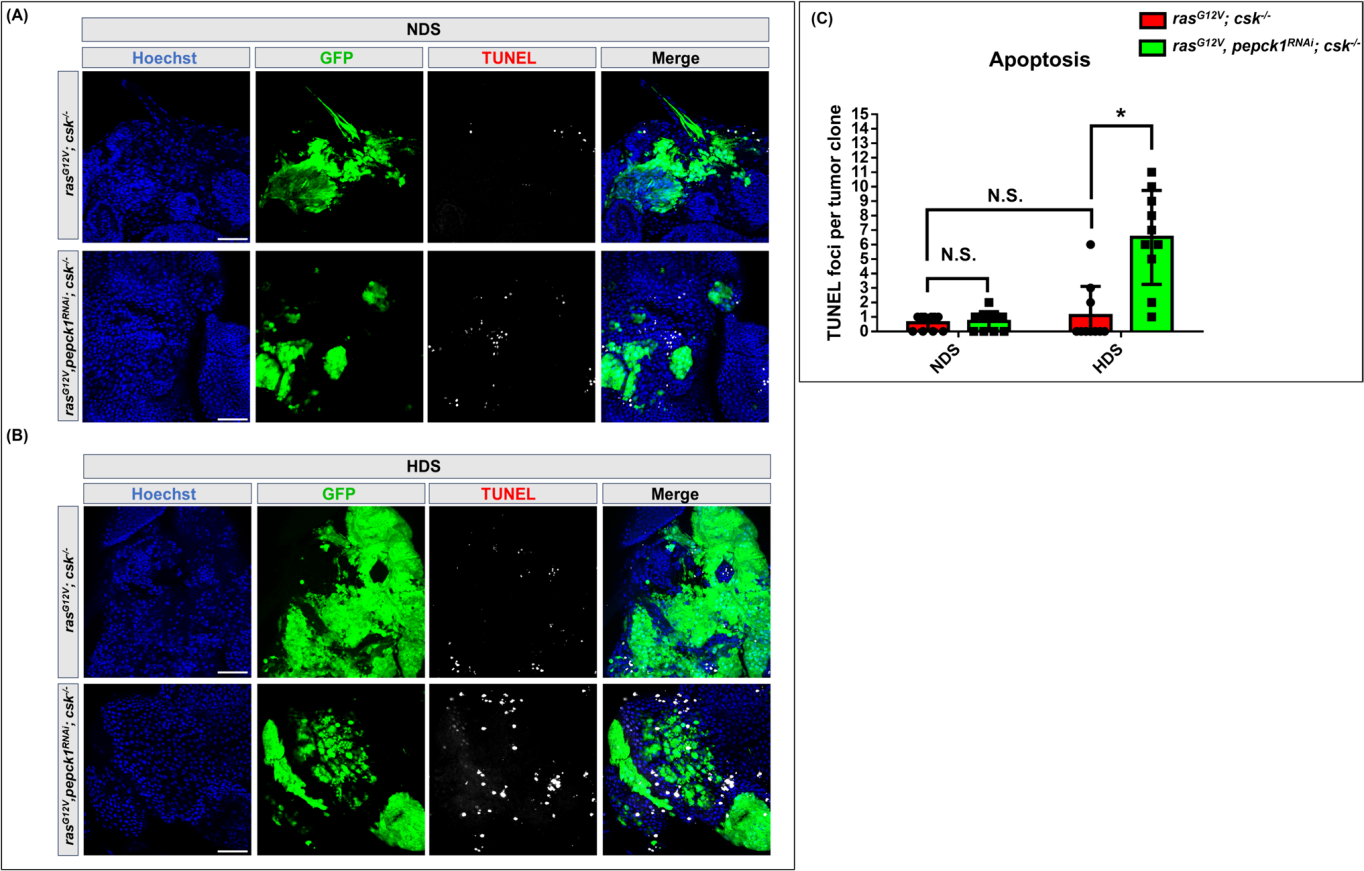
Knockdown of *pepck1* induces apoptosis in tumor cells. **A**, **B** Eye discs from *ras*^*G12V*^*; csk*^*−/−*^ and *ras*^*G12V*^*, pepck1*^*RNAi*^*; csk*^*−/−*^ female *Drosophila* 3rd instar larvae, fed NDS or HDS, with GFP-labeled tumor cells (green) and TUNEL staining (white). Scale bar: 20 μm. **C** Quantification of TUNEL foci in tumor cells per tumor clone; n = 6 female eye discs per group. Results are shown as mean ± SD. Asterisk indicates a statistically significant difference via two-way ANOVA with paired control (*P < 0.05). *GFP* green fluorescent protein, *Blue* Hoechst, *HDS* high dietary sugar, *NDS* normal dietary sugar, *N.S.* not significant, *SD* standard deviation

HDS induces evasion of apoptosis in tumor cells [6, 18]. Therefore, we investigated whether upregulated *pepck1* decreases apoptosis in HDS-induced tumor cells. Using the terminal deoxynucleotidyl transferase dUTP nick end labeling (TUNEL) assay, which identifies cells undergoing programmed cell death by detecting DNA fragmentation, we observed a similar number of TUNEL-positive foci in tumors under both NDS and HDS conditions, which is consistent with previous findings from our group [18] (Fig. 5A–C). Moreover, *pepck1* knockdown in HDS-induced Ras/Src tumors significantly increased apoptosis in tumor cells (Fig. 5A–C). These results indicate that the anti-apoptotic effect observed in HDS-induced tumors can be reversed by downregulation of *pepck1*. These findings suggest that HDS-induced upregulation of PEPCK1 contributes to tumor cell evasion of apoptosis.

### Knockdown of *pepck1* reduces HDS-induced genome instability in tumor cells

**Fig. 6:**
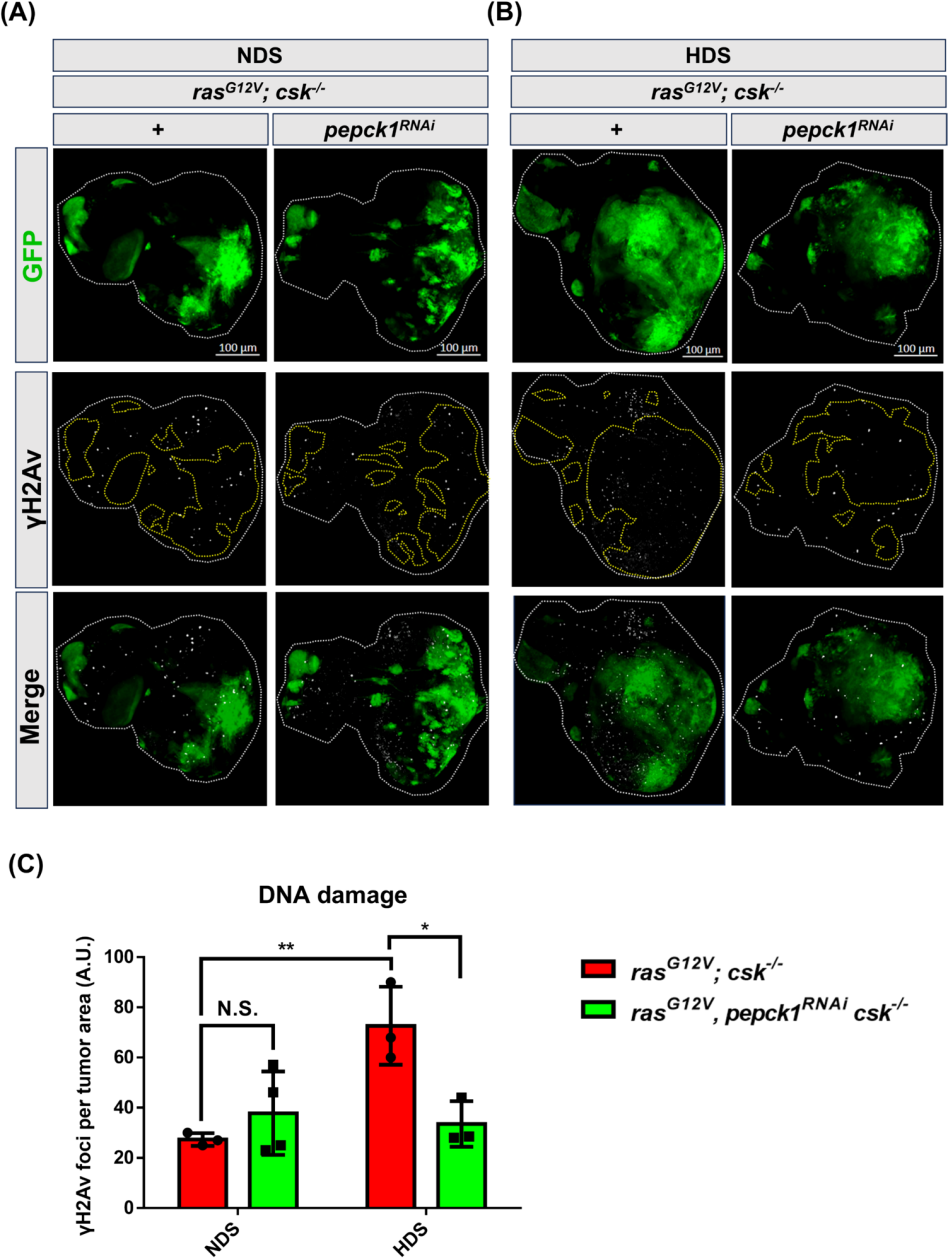
Knockdown of *pepck1* reduces HDS-induced DNA damage in tumor cells. **A**, **B** Eye discs from *ras*^*G12V*^*; csk*^*−/−*^ and *ras*^*G12V*^*, pepck1*^*RNAi*^*; csk*^*−/−*^ 3rd instar female *Drosophila* larvae, fed NDS or HDS, with GFP-labeled tumor cells (green) and γH2AX immunostaining (white). Scale bar: 100 μm. **C** Quantification of γH2AX foci in tumor cells; n = 6 female eye discs per group. Results are shown as mean ± SD. Asterisks indicate statistically significant differences via two-way ANOVA with paired control (*P < 0.05; **P < 0.01). *GFP* green fluorescent protein, *HDS* high dietary sugar, *NDS* normal dietary sugar, *N.S.* not significant, *SD* standard deviation

Genome instability significantly contributes to the development of tumors [8]. Moreover, genome instability plays a crucial role in the development and progression of tumors in *Drosophila* [37]. Notably, a previous report investigated the link between *pepck1* and chromosomal instability in a *Drosophila brat* tumor explant model [38]. Therefore, we investigated whether upregulation of *pepck1* promotes HDS-induced genome instability in tumor cells. Consistent with our previous findings[18], the number of γ-H2AX foci, a marker for double-stranded DNA breaks and DNA damage, was increased in tumor cells under HDS conditions. Importantly, *pepck1* knockdown reduced the number of γ-H2AX foci in HDS-induced tumor cells (Fig. 6A–C). Therefore, *pepck1* knockdown enhances genome stability in tumor cells under HDS, suggesting that *pepck1* increases genome instability in HDS-induced tumor cells.

### Hydrazinium sulfate further decreases HDS-induced developmental delay and increases survival of tumor-bearing animals with *pepck1* knockdown

**Fig. 7:**
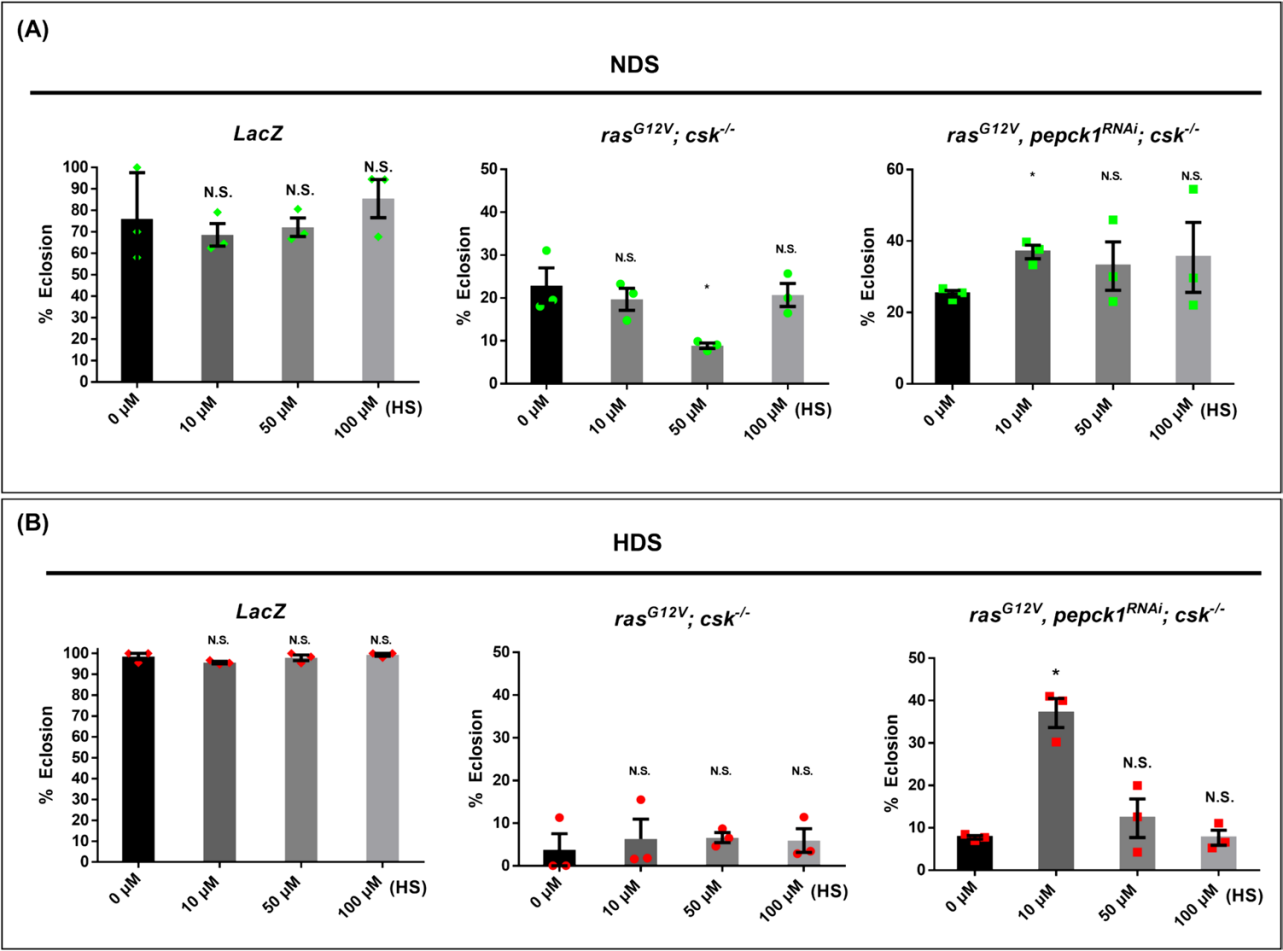
Hydrazinium sulfate (HS), an inhibitor of PEPCK, enhances the survival of tumor-bearing animals with *pepck1* knockdown. *Drosophila* larvae with the following genotypes were fed either NDS or HDS and used in the experiments: *lacZ, and ras*^*G12V*^*; csk*^*−/−*^* and ras*^*G12V*^*; pepck1*^*RNAi*^*; csk*^*−/−*^. A total of 200 combined male and female larvae per genotype were used for each experiment. **A** Eclosion rates of animals fed NDS with treatments of 0 µM, 10 µM, 50 µM, or 100 µM HS. **B** Eclosion rates of animals fed HDS with treatments of 0 µM, 10 µM, 50 µM, or 100 µM HS. Results are shown as mean ± SD. Asterisks indicate statistically significant differences via two-way ANOVA with paired controls (*P < 0.05). *HDS* high dietary sugar, *HS* hydrazinium sulfate, *NDS* normal dietary sugar, *N.S.* not significant, *SD* standard deviation

To assess whether pharmacological targeting of PEPCK1 offers a preventive and therapeutic strategy against HDS-induced tumorigenesis in vivo, we employed hydrazinium sulfate (HS), an inhibitor of PEPCK, in an attempt to suppress tumor progression by inhibiting PEPCK1 [38]. The mechanism of action for HS involves blocking the binding site of the PEPCK enzyme on oxaloacetate (OAA), thereby impeding the conversion of OAA to phosphoenolpyruvate (PEP) [39]. Additionally, HS reduces gluconeogenesis by inhibiting PEPCK, which decreases both pyruvate carboxylase (PC) activity and the transport of pyruvate to the mitochondria [40]. Initially, we investigated whether inhibiting PEPCK1 could reduce developmental delay and lethality induced by HDS in tumor-bearing animals. We evaluated the eclosion rates of wild-type (*lacZ*), tumor-bearing animals (*ras*^*G12V*^*; csk*^*−/−*^), and tumor-bearing animals with *pepck1* knockdown (*ras*^*G12V*^*, pepck1*^*RNAi*^*; csk*^*−/−*^), respectively, that were treated with HS at concentrations of 0 µM, 10 µM, 50 µM, and 100 µM. In our study of survival rates of tumor-bearing animals, we observed that HS is non-toxic to wild-type (lacZ) *Drosophila*, regardless of whether they were fed NDS or HDS (Fig. 7A, B). Additionally, we observed a significant increase in the survival rate of HDS-fed tumor-bearing animals with *pepck1* knockdown (*ras*^*G12V*^*, pepck1*^*RNAi*^*; csk*^*−/−*^) treated with 10 µM HS, compared to those receiving no HS (0 µM treatment) (Fig. 7). Moreover, under NDS, the survival rates of tumor-bearing animals (*ras*^*G12V*^*; csk*^*−/−*^) decreased with 50 µM HS treatment compared to those not treated with HS (0 µM treatment) (Fig. 7). In contrast, the survival rate of NDS-fed tumor-bearing animals with *pepck1* knockdown (*ras*^*G12V*^*, pepck1*^*RNAi*^*; csk*^*−/−*^) improved with a 10 µM HS treatment (Fig. 7). Thus, the combined treatment with 10 µM HS and *pepck1* knockdown significantly reduces the lethality of tumor-bearing animals. Overall, these results indicate that inhibition of PEPCK by HS effectively enhances the survival of tumor-bearing animals with pepck1 knockdown under conditions of HDS.

## Discussion

Globally, more than 650 million adults are clinically obese, with excessive consumption of added sugars significantly contributing to metabolic disorders and associated cancers [41, 42]. Better understanding of the complex relationship between diet and cancer at molecular, cellular, and organismal levels, by exploring the role of metabolic alterations in promoting HDS-induced cancer progression, is crucial for the prevention and treatment of diet-associated cancers. In this study, we employed a range of techniques, including bioinformatics, and discovered that *pepck1* is surprisingly upregulated by HDS during tumor progression. Interestingly, *pepck1* is directly downregulated by HP1a-mediated heterochromatin formation in wild-type adult flies. Our previous studies have consistently shown that HP1a-mediated heterochromatin formation is a significant epigenetic factor in suppressing tumor development induced by a high sugar diet, while HDS itself downregulates HP1a during tumor progression [18]. Therefore, HDS-induced reduction of HP1a-mediated heterochromatin formation may explain aberrant epigenetic upregulation of *pepck1* during tumor progression induced by a diet that is high in sugar [18]. However, it remains unclear whether HDS-mediated increase in *pepck1* gene expression in tumors is due to direct epigenetic modulation by HP1a-mediated heterochromatin formation. To address this in future studies, HP1a-mediated heterochromatin formation should be promoted using the *ras*^*G12V*^*, HP1a; csk*^*−/−*^ fly line [18] and tested to determine whether the *pepck1* mRNA levels are decreased in *ras*^*G12V*^*, HP1a; csk*^*−/−*^ tumors under HDS. Furthermore, to demonstrate that increased *pepck1* is due to epigenetic changes, a ChIP assay of the *pepck1* gene from Ras/Src-tumors under NDS and HDS should be performed. Moreover, PEPCK1, a crucial cytosolic enzyme in gluconeogenesis, is upregulated in many human cancers, indicating its significant role in cancer progression [15]. However, to our knowledge, this study is the first to identify the paradoxical upregulation of PEPCK1 by HDS during tumor progression. Thus, we aimed to determine the role of PEPCK1 in HDS-induced cancer progression by utilizing the *Drosophila* Ras/Src cancer model, which shares many hallmarks of human cancer progression [43]. Our research uncovered new mechanisms through which the abnormal enhancement of PEPCK1 accelerates tumor growth in response to a high sugar diet. This involves the activation of the wingless/Wnt and mTOR/TOR signaling pathways, suppression of apoptosis, increased genome instability, and the reprogramming of carbohydrate metabolism to result in increased glucose uptake and trehalose levels. These insights deepen our understanding of the complex link between diet and cancer, highlighting potential points of intervention for prevention and treatment of cancers linked to metabolic disorders.

Numerous cancer types exhibit alterations in key enzymes involved in gluconeogenesis that contribute to reprogramming of metabolic pathways or induce tumor progression [44–46]. These adjustments increase metabolic flexibility and permit the incorporation of non-carbohydrate substances in biosynthesis and the modification of glucose flows to enhance antioxidant production. Specifically, PEPCK1 promotes colon cancer growth by increasing gluconeogenesis metabolites through phosphoenolpyruvate and pyruvate production [14]. Likewise, *pepck2* gene expression is increased in thyroid, bladder, breast, kidney, and non-small-cell lung cancer [44]. Interestingly, prior research has suggested that PEPCK1 plays tumor suppressor role in liver cancer [47, 48]. These findings highlight the complex roles of PEPCK1 and PEPCK2 in cancer progression across various types of human cancer, despite the tumor-suppressive function of PEPCK1 in hepatocellular carcinoma. Our research reveals that enzymes such as *pepck1*, *pepck2*, and *Ldh* are overexpressed during tumor progression, after being triggered by HDS. Furthermore, aberrant upregulation of *pepck1* leads to elevated trehalose synthesis and glucose uptake. Thus, we have uncovered new mechanisms by which PEPCK1 facilitates reprogramming of carbohydrate metabolism during HDS-induced tumor progression.

In this study, we discovered that knockdown of *pepck1*, but not *pepck2*, reduces tumor progression induced by HDS. We consistently found that overexpression of *pepck2* does not enhance tumorigenesis under HDS conditions. Interestingly, even overexpression of *pepck1* does not increase tumor progression under NDS or HDS. These results suggest that the maximum potential of PEPCK1 to promote tumor growth may already be reached under HDS, resulting in no additional effect of overexpression. In other words, HDS conditions likely maximize *pepck1* expression and its associated effects in promoting tumor growth in the Ras/Src cancer model. Importantly, PEPCK1 does not appear to influence tumor progression under NDS, highlighting its role as specific to the HDS context. Conversely, PEPCK2 does not significantly affect tumor progression, in either promotion or inhibition, or under either HDS or NDS conditions, indicating a possible specificity in the function of PEPCK1 and PEPCK2 related to cancer type and/or dietary context combination.

In this study, we demonstrated that the knockdown of *pepck1* in Ras/Src tumor cells suppresses HDS-induced genome instability (Fig. 6). Notably, the HDS-induced increase in γ-H2AX foci and the genome instability suppression effect from *pepck1* knockdown are observed across the entire tissue, not just in the tumor cells. This finding aligns with our previous observation of non-cell-autonomous DNA damage occurring in non-tumor cells adjacent to tumor cells [18]. Furthermore, a similar non-cell-autonomous effect was observed concerning HDS-induced evasion of apoptosis and the influence of *pepck1* knockdown within tumor cells (Fig. 5) [18]. The molecular mechanisms underlying these non-cell-autonomous effects are not yet fully understood and are currently under investigation. In *Drosophila*, apoptotic cells have been shown to produce non-cell-autonomous signals, including wingless, Dpp, and TNF, which can induce compensatory apoptosis in neighboring cells within the wing imaginal disc [49]. Additionally, chromosomal instability has been implicated in promoting non-cell-autonomous cancer progression through interactions with the immune system in both mouse cancer models and human breast cancer cells [50]. Future studies are needed to elucidate the mechanisms driving non-cell-autonomous apoptosis and genome instability effects on HDS-induced tumor progression.

Here, we found that targeted pharmacological inhibition of PEPCK1 with HS significantly enhances the survival of tumor-bearing animals with *pepck1* knockdown under NDS and HDS. Our findings align with a previous study that also demonstrated the effectiveness of HS in inhibiting PEPCK1 and suppressing brain tumor growth in *Drosophila* [38]. Interestingly, the survival of *ras*^*G12V*^*, pepck1*^*RNAi*^*; csk*^*−/−*^ animals is increased compared to *ras*^*G12V*^*; csk*^*−/−*^ animals under HDS (Fig. 1G), while HS treatment did not affect the survival of *ras*^*G12V*^*; csk*^*−/−*^ animals under HDS (Fig. 7B; middle panel). It is likely that the concentrations of 10 µM, 50 µM, and 100 µM HS may not be high enough to inhibit the upregulated levels of PEPCK1 in *ras*^*G12V*^*; csk*^*−/−*^ animals under HDS. Future studies are necessary to test whether feeding HS alone at a higher concentration, such as 10 mM of HS mixed in the fly diet [38], is sufficient to enhance the survival of *ras*^*G12V*^*; csk*^*−/−*^ animals. Moreover, combining 10 µM HS treatment with *pepck1* knockdown significantly decreases the lethality in tumor-bearing animals under both NDS and HDS (Fig. 7A, B; right panels). It is likely that 10 µM HS treatment only improves survival in animals with *ras*^*G12V*^*, pepck1*^*RNAi*^*; csk*^*−/−*^ tumors due to the effective reduction of PEPCK1 levels by *pepck1* RNAi, which allows 10 µM HS to sufficiently inhibit PEPCK1 in this situation. Furthermore, future studies should determine whether the effect of HS on the increased survival of *ras*^*G12V*^*, pepck1*^*RNAi*^*; csk*^*−/−*^ animals is due to tumor suppression rather than systemic effects independent of tumor growth by measuring the tumor size in Fig. 7.

**Fig. 8:**
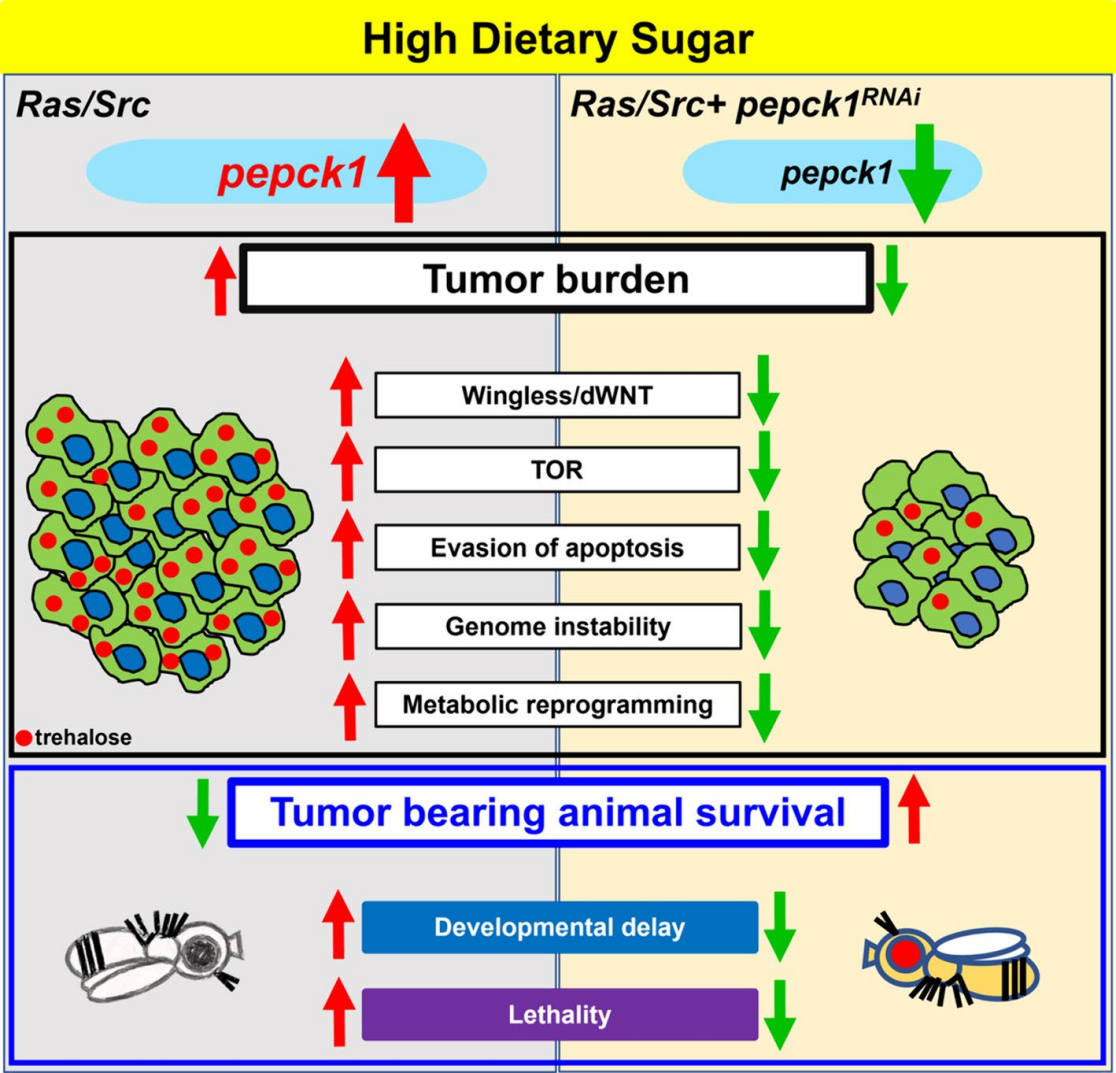
High sugar diet induces upregulation of *pepck1*, which promotes cancer progression by acivating wingless/dWnt and TOR signaling, decreasing apoptosis, inducing genome instability, and reprogramming carbohydrate metabolism. In the presence of HDS, tumor cells upregulate PEPCK1 to increase tumor burden and decrease the survival rate of tumor-bearing animals. Additionally, the heterochromatin formed via an HP1a-mediated process directly suppresses *pepck1* expression in tumor cells. Mechanistically, PEPCK1 facilitates HDS-induced tumor progression by elevating wingless expression and TOR signaling, suppressing apoptosis, inducing genome instability, and reprogramming carbohydrate metabolism through increased glucose uptake and elevated trehalose levels

Overall, our research adds to the understanding of the molecular, cellular, and organismal mechanisms through which upregulation of PEPCK1 leads to HDS-aggravated tumor progression. These findings deepen our understanding of the intricate connections between diet and cancer through cellular and molecular processes, revealing new potential targets for both preventing and treating cancers linked to metabolic disorders, as well as mitigating the impact of dietary factors on cancer progression (Fig. 8).

## Materials and methods

### Fly stocks

The following fly strains were obtained as gifts from Dr. Ross Cagan at University of Glasgow: (1) *ey(3.5)-FLP1; act* > *y* +  > *gal4,UAS-GFP; FRT82B, tub-gal80*, (2) *UAS-lacZ; FRT*^*82B*^, and (3) *UAS-ras*^*G12V*^*; FRT*^*82B*^*, csk*^*Q156*^*/TM6B*. Additionally, the following strains were constructed in our lab: (4) *UAS-ras*^*G12V*^*, pepck1*^*RNAi*^*; FRT*^*82B*^*, csk*^*Q156*^*/TM6B*, (5) *UAS-ras*^*G12V*^*, pepck2*^*RNAi*^*; FRT*^*82B*^*, csk*^*Q156*^*/TM6B,* (6) *UAS-ras*^*G12V*^*, pepck1::mcherry; FRT*^*82B*^*, csk*^*Q156*^*/TM6B,* and (7) *UAS-ras*^*G12V*^*, pepck2; FRT*^*82B*^*, csk*^*Q156*^*/TM6B*. Virgin females of the *ey(3.5)-FLP1; act* > *y* +  > *gal4,UAS-GFP; FRT82B, tub-gal80* strain were crossed with flies carrying various constructs to generate tumor-bearing animals with knockdown or overexpression of *pepck1* and *pepck2*. The specific strains utilized included: *UAS-pepck1*^*RNAi*^ (VDRC 20529), *UAS-pepck2*^*RNAi*^ (VDRC 13929), *UAS-pepck1::mcherry* (cross of strains 4, 5, and 6 listed above) and *UAS-pepck2* (strain 7 above)*,* both of which were generated by our team for this research.

### Fly cultures, immunofluorescence, quantitative RT-PCR, RNA in situ hybridization, Glucose and trehalose assay, ChIP assay and TUNEL assay

The detailed procedure is described in Supplementary Materials and Methods.

### Geneswitch inducible system

To activate specific gene expression, adult flies were maintained on a diet that included food mixed with 300 μM RU486 or with solvent (ethanol) only as a control. Following four days of feeding, the flies were homogenized and their cells were lysed to extract RNA.

### Statistical analysis

The data presented in this study were derived from a minimum of three separate experiments and were analyzed using PRISM 6 software (GraphPad, San Diego, CA). The results were expressed as the mean ± standard deviation (SD). Statistical significance was assessed using two-way ANOVA or Student’s t-test, as indicated in the respective figure legends. A *P*-value of less than 0.05 was considered statistically significant.

## Supplementary Information

Below is the link to the electronic supplementary material.

Supplementary file1 (DOCX 5188 KB)

Supplementary file2 (DOCX 28 KB)

## Data Availability

All data are contained within the manuscript; all raw data from our study are available from the corresponding author upon reasonable request.
