# Supplementary material for: High sugar diet promotes tumor progression paradoxically through aberrant upregulation of pepck1
Source: Cell Mol Life Sci. 2024 Sep 11;81(1):396. doi: 10.1007/s00018-024-05438-2 (PMC11390995; doi:10.1007/s00018-024-05438-2)
Supplement: Supplementary file 1 — Supplementary file1 (DOCX 5188 KB) [file 18_2024_5438_MOESM1_ESM.docx]

**Supplementary Figures for**

**High sugar diet promotes tumor progression paradoxically through aberrant upregulation of *pepck1***

**Che-Wei Chang^1,2^, Yu-Hshun Chin^*,2^,** **Meng-Syuan Liu^*,2^, Yu-Chia Shen^2^ and Shian-Jang Yan^#,1,2^**

^1^Institute of Basic Medical Sciences, College of Medicine, National Cheng Kung University, No. 1, University Road, Tainan City, Taiwan

^2^Department of Physiology, College of Medicine, National Cheng Kung

University, No. 1, University Road, Tainan City, Taiwan

*These authors have contributed equally to this work.

^#^Corresponding author. Tel: +886 62353535 ext 5437;

E-mail: [johnyan@gs.ncku.edu.tw](mailto:johnyan@gs.ncku.edu.tw)


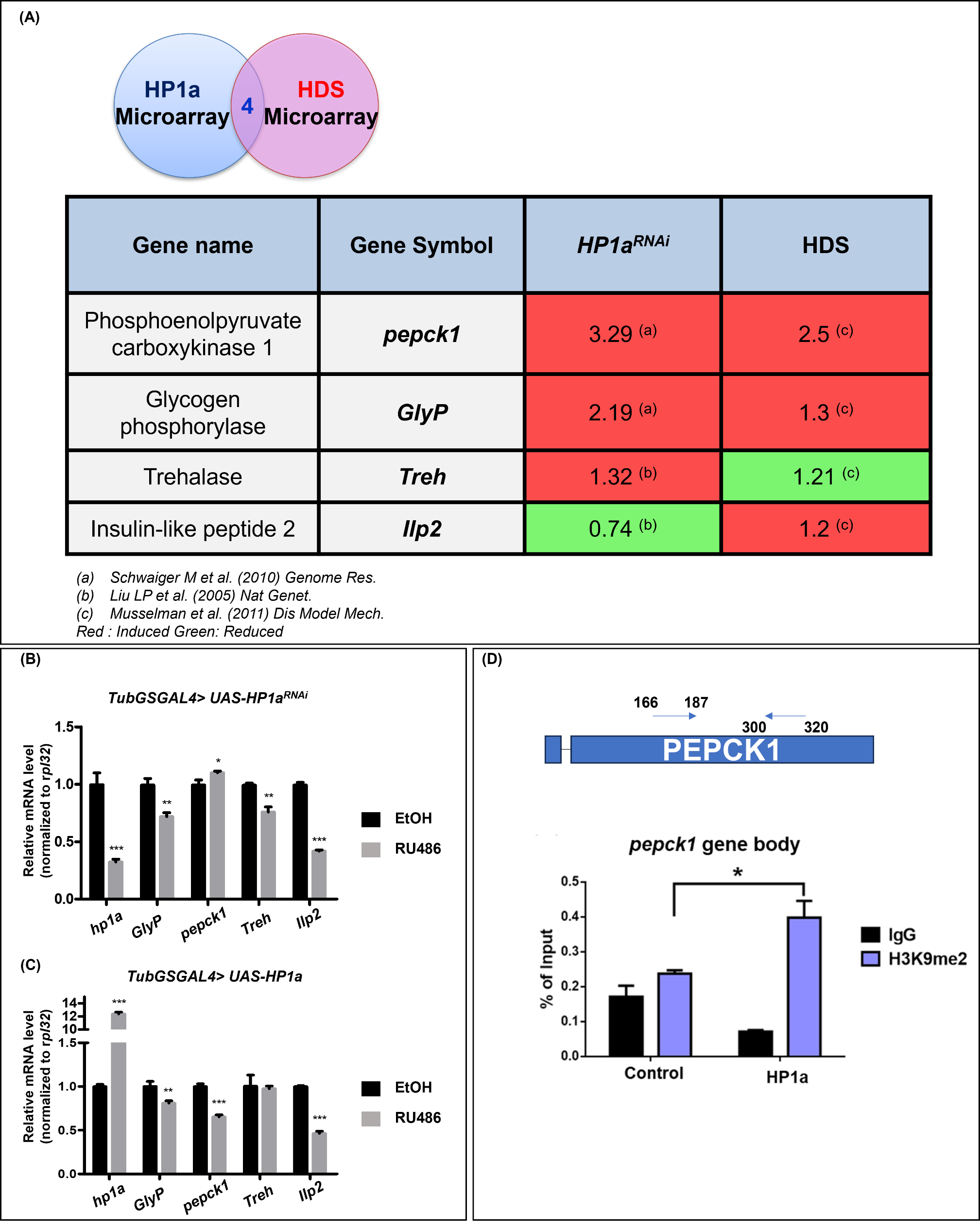


**Supplementary Fig. 1.** **HP1a-mediated heterochromatin directly interacts with the *pepck1* gene, resulting in downregulation of *pepck1* expression.**

(A) Identification of candidate metabolic genes by integrating *HP1a^RNAi^* microarray and HDS microarray databases. (B-C) Relative mRNA expression levels of *HP1a* and several metabolic genes. Male flies of *tubGSgal4*>*UAS-HP1a* and *tubGSgal4*>*UAS-HP1a^RNAi^* were treated with fly food mixed with EtOH only (control) or RU486 for 4 days. All genes were normalized to *rpl32*. Total RNA was extracted from 10 adult flies. (D) ChIP assays of the *pepck1* gene body or promoter region were performed using IgG (control) or an H3K9me2 antibody. A total of 100 male flies were used in each experiment. Results are shown as mean ± SD. Asterisks indicate statistically significant differences via Student’s t-test (**P* < 0.05; ***P* < 0.01; ****P* < 0.001). ChIP, chromatin immunoprecipitation; EtOH, ethanol; HDS, high dietary sugar; NDS, normal dietary sugar; SD, standard deviation.


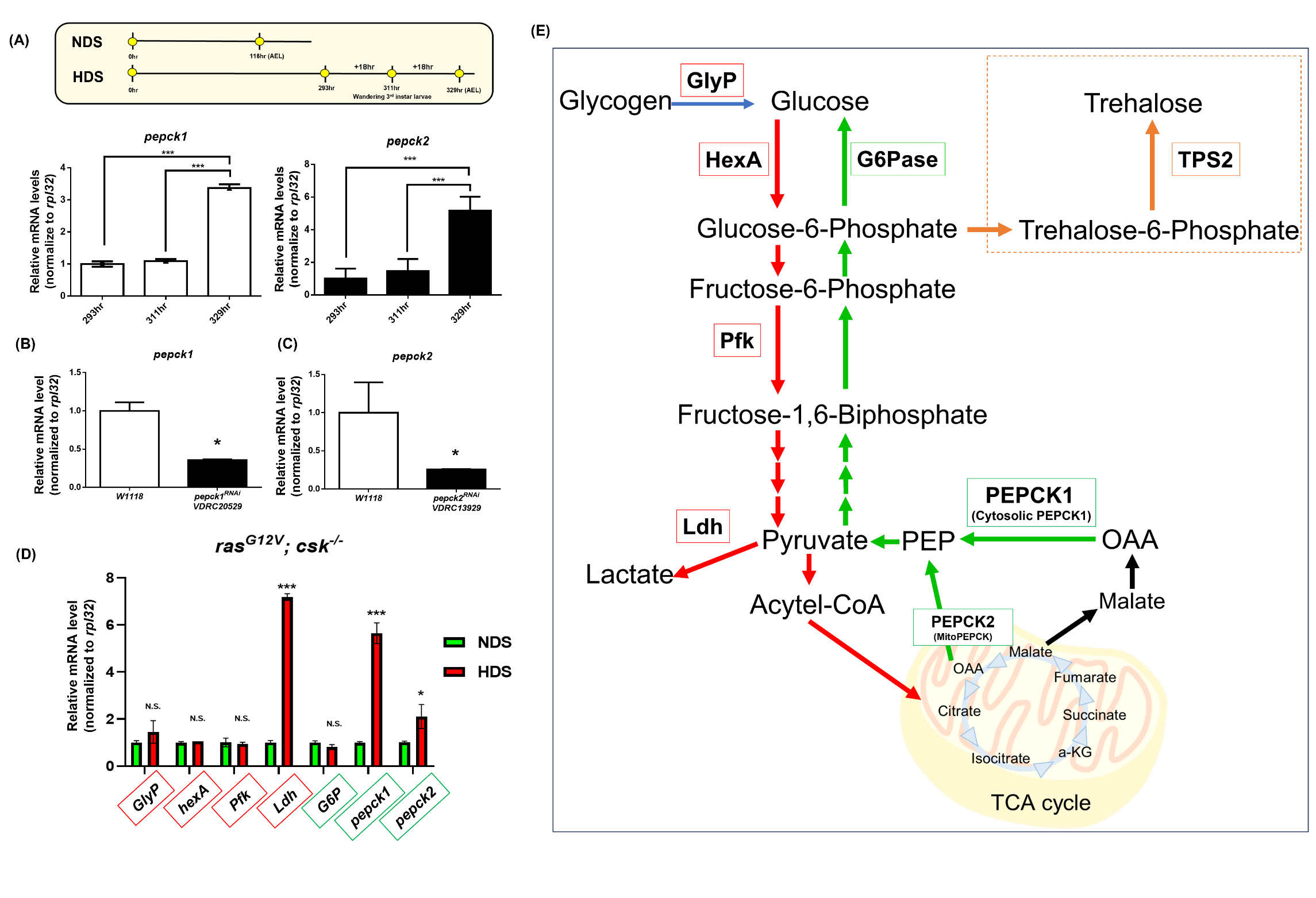
**Supplementary Fig. 2. *pepck1* and *2* are upregulated during HDS-induced tumor progression.**

(A) Experimental scheme: *ras^G12V^; csk^-/-^* tumor-bearing animals were fed HDS. The relative levels of *pepck1/2* mRNA were assessed based on RNA extracted from 30 eye discs from combined 3rd instar male and female larvae (n=15) fed HDS (1.0M M sucrose diet). Results were normalized to *rpl32* (B, C) Total RNA was extracted from 10 non-tumor combined 3rd instar male and female larvae of *DaGAL4* crossed with *UAS-pepck1^RNAi^* and *UAS-pepck2^RNAi^*. (D) Total RNA was extracted from combined 30 tumor-bearing *ras^G12V^; csk^-/-^*3rd instar male and female larvae fed HDS. All gene expression was normalized to *rpl32*. (E) Simplified depiction of glucose metabolism pathways and related genes. Results are shown as mean ± SD. Asterisks indicate statistically significant differences via Student’s t-test (**P* < 0.05; ***P* < 0.01; ****P* < 0.001). G6Pase, glucose-6-phosphatase; GlyP, glycogen phosphorylase; HexA, hexokinase; HDS, high dietary sugar; Ldh, lactate dehydrogenase; NDS, normal dietary sugar; N.S., not significant; Pfk, phosphofructokinase; SD, standard deviation; TPS2, trehalose-6-phosphate phosphatase.


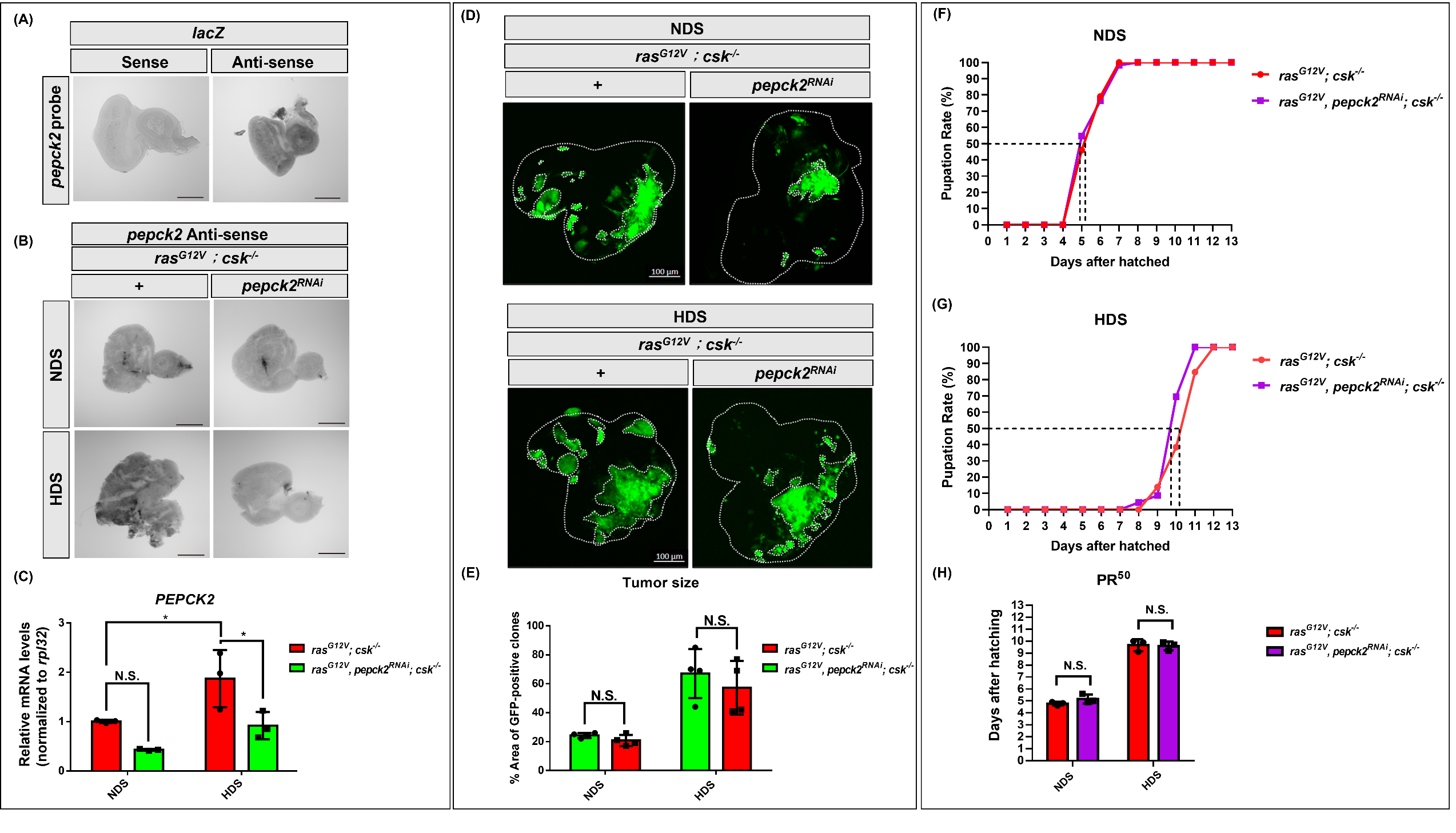


**Supplementary Fig. 3.** **Knockdown of *pepck2* in tumor cells does not result in significant alterations in tumor growth, developmental delay, or lethality in Ras/Src tumor-bearing animals under HDS.**

(A) mRNA expression patterns of *pepck2* via DIG-labeled *pepck2* sense (negative control) or antisense RNA probes in WT male eye discs. (B) mRNA expression patterns of *pepck2* via DIG-labeled *pepck2* antisense RNA probes in eye discs of *ras^G12V^; csk^-/-^* and *ras^G12V^, pepck2^RNAi^; csk^-/-^* male *Drosophila* larvae under NDS or HDS. (C) Relative levels of *pepck2* mRNA via RNA extracted from 30 eye discs of combined 3rd instar male and female larvae (n=15) fed HDS. Results were normalized to *rpl32* (D) Eye discs from *ras^G12V^; csk^-/-^* and *ras^G12V^, pepck2^RNAi^; csk^-/-^* 3rd instar female larvae fed NDS or HDS with GFP-labeled tumor cells. (E) Percentage of GFP-positive tumor area was normalized to the total area of the eye disc from female *Drosophila* fed NDS or HDS; n=6 eye discs per group. Scale bar: 100 µm. (F) Pupation rates of combined male and female animals fed NDS. (G) Pupation rates of combined male and female animals fed HDS. (H) Number of days (PR^50^) until pupation rate reached 50% among combined male and female tumor-bearing animals. Results are shown as mean ± SD. Asterisks indicate statistically significant differences via two-way ANOVA with relevant paired controls (**P* < 0.05). GFP green fluorescent protein; HDS, high dietary sugar; NDS, normal dietary sugar; N.S., not significant; SD, standard deviation.


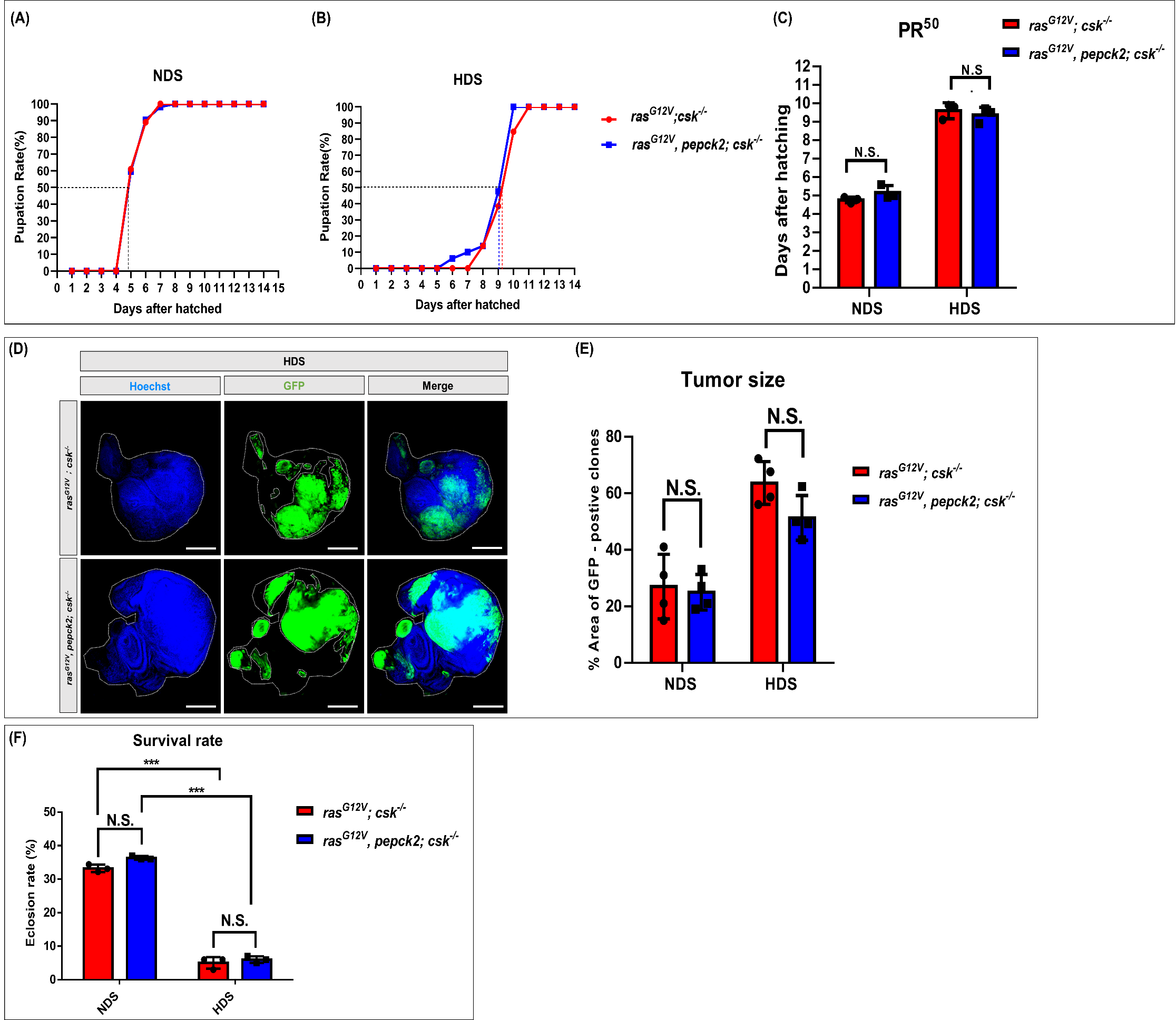
**Supplementary Fig. 4. Overexpression of *pepck2* does not alter the developmental delay and lethality of Ras/Src tumor-bearing flies.**

*Drosophila* with the following genotypes were used in the experiments: *lacZ* (control), *ras^G12V^; csk^-/-^* (tumor-bearing), and *ras^G12V^, pepck2; csk^-/-^* (tumor-bearing with *pepck2* overexpression). (A) Pupation rates of combined male and female animals fed a 0.15 M sucrose diet (NDS). (B) Pupation rates of combined male and female animals fed a 0.75 M sucrose diet (HDS). (C) Number of days (PR^50^) until pupation rate reached 50% among combined male and female tumor-bearing animals. (D) GFP-labeled tumor cells (green) and Hoechst staining (blue) of 3rd instar larvae eye discs from female *Drosophila* of strain *ras^G12V^; csk^-/-^* and *ras^G12V^, pepck2; csk^-/-^* that were fed HDS. Scale bar: 100 μm. (E) Percentage of GFP-positive tumor area was normalized to the total area of the eye disc from female *Drosophila* fed NDS or HDS; n=6 eye discs per group. (F) Eclosion rates of *ras^G12V^; csk^-/-^* and *ras^G12V^, pepck2; csk^-/-^* combined male and female larvae fed NDS or HDS. Results are shown as mean ± SD. Asterisk indicates a statistically significant difference via two-way ANOVA with paired control (****P* < 0.001). GFP, green fluorescent protein; HDS, high dietary sugar; NDS, normal dietary sugar; N.S., not significant, SD, standard deviation.


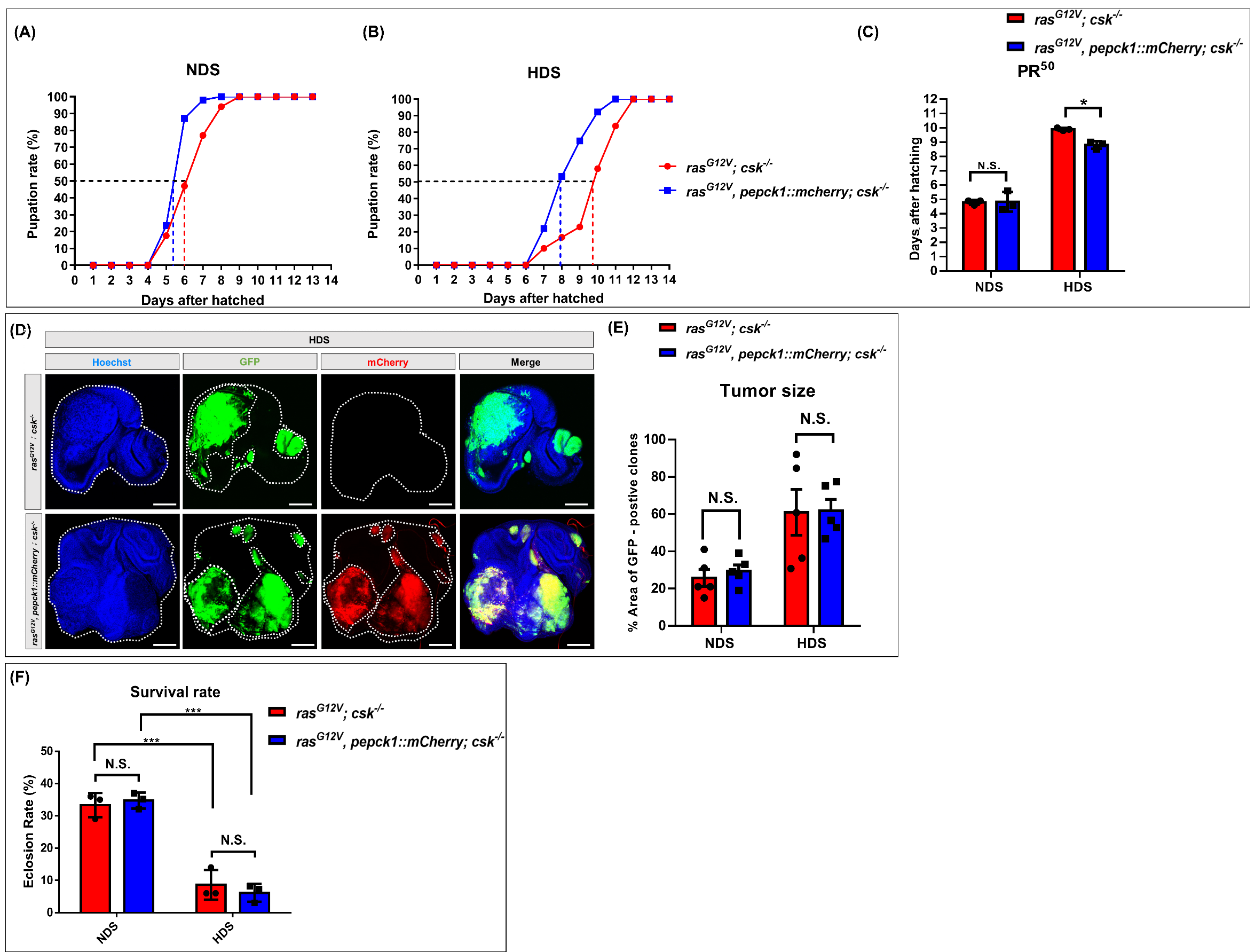
**Supplementary Fig. 5. Overexpression of *pepck1* slightly reduces developmental delay but has no effect on lethality or HDS-induced tumor growth of Ras/Src tumor-bearing animals fed HDS.**

*Drosophila* with the following genotypes were generated: *ras^G12V^; csk^-/-^* (tumor-bearing) and *ras^G12V^, pepck1::mCherry; csk^-/-^* (tumor-bearing with overexpression of *pepck1*, tagged with mCherry). (A) Pupation rates of combined male and female larvae fed a 0.15 M sucrose diet (NDS). (B) Pupation rates of combined male and female larvae fed a 0.75 M sucrose diet (HDS). (C) Number of days (PR^50^) until pupation rate reached 50% among combined male and female tumor-bearing animals. (D) GFP-labeled tumor cells (green), pepck1::mCherry expression (red), and Hoechst staining (blue) of 3rd instar larvae eye discs of *ras^G12V^; csk^-/-^* and *ras^G12V^, pepck1::mCherry; csk^-/-^* female *Drosophila* larvae fed NDS or HDS. Scale bar: 100 μm. (E) Percentage of GFP-positive area normalized to total eye disc area; n=6 eye discs per group. Quantification of fluorescence intensity in tumor cells from female *Drosophila* larvae fed HDS. Results are shown as mean ± SD of individual eye discs. (F) Eclosion rates of *ras^G12V^; csk^-/-^* and *ras^G12V^, pepck1::mCherry; csk^-/-^* combined male and female *Drosophila* fed NDS or HDS. Asterisk indicates a statistically significant difference via two-way ANOVA with paired control (****P* < 0.001). GFP, green fluorescent protein; HDS, high dietary sugar; NDS, normal dietary sugar; N.S., not significant, SD, standard deviation.
