# Supplementary material for: High sugar diet promotes tumor progression paradoxically through aberrant upregulation of pepck1
Source: Cell Mol Life Sci. 2024 Sep 11;81(1):396. doi: 10.1007/s00018-024-05438-2 (PMC11390995; doi:10.1007/s00018-024-05438-2)
Supplement: Supplementary file 2 — Supplementary file2 (DOCX 28 KB) [file 18_2024_5438_MOESM2_ESM.docx]

**Supplementary Materials and Methods for**

**High sugar diet promotes tumor progression paradoxically through aberrant upregulation of *pepck1***

**Che-Wei Chang^1,2^, Yu-Hshun Chin^*,2^,** **Meng-Syuan Liu^*,2^, Yu-Chia Shen^2^ and Shian-Jang Yan^#,1,2^**

^1^Institute of Basic Medical Sciences, College of Medicine, National Cheng Kung University, No. 1, University Road, Tainan City, Taiwan

^2^Department of Physiology, College of Medicine, National Cheng Kung

University, No. 1, University Road, Tainan City, Taiwan

*These authors have contributed equally to this work.

^#^Corresponding author. Tel: +886 62353535 ext 5437;

E-mail: [johnyan@gs.ncku.edu.tw](mailto:johnyan@gs.ncku.edu.tw)

**Supplementary Materials and Methods**

**Fly cultures**

Flies were maintained at 25 ℃ and cultured on Bloomington semi-defined medium, as described by the Bloomington Stock Center. To investigate the effects of high dietary sugar on tumor progression, tumor-bearing animals were fed different diets. For normal dietary sugar (NDS) conditions, the agar medium consisted of 5 g agar, 40 g brewer’s yeast, 10 g yeast extract, 10 g peptone, 25.5 g sucrose, and water up to 500 ml. For high dietary sugar (HDS) conditions, the agar medium comprised 5 g agar, 40 g brewer’s yeast, 10 g yeast extract, 10 g peptone, 128.25 g sucrose, and water up to 500 ml. Unless otherwise specified, *Drosophila* fed NDS and HDS were dissected on day 5 and day 8 after egg laying (AEL), respectively.

**Immunofluorescence and quantification**

Larval eye discs were dissected in phosphate-buffered saline (PBS) and then fixed for 10 minutes with 4% paraformaldehyde (PFA) in PBS. Following fixation, the discs were washed three times in PBST (PBS containing 0.3% Triton X-100). Subsequently, the discs were incubated with primary antibodies and then washed again before being incubated with secondary antibodies along with Hoechst stain (Thermo Fisher; 1:10000). Primary antibodies used included mouse monoclonal anti-wingless (4D4, Development Hybridoma Bank; 1:500) and/or rabbit anti-*Drosophila* phospho-RpS6 (pS6) antibody (provided by Dr. Aurelio Teleman at German Cancer Research Center [DKFZ]; 1:300), diluted in PBST with normal goat serum (NGS). After secondary antibody incubation, which involved goat anti-rabbit/anti-mouse conjugated to Alexa Fluor-594, the samples were mounted with Vectashield Antifade Mounting Medium (Vector Laboratories, USA). Finally, the mounted samples were examined using a confocal microscope (Carl Zeiss LSM780) located at the Institute of Biotechnology, National Cheng Kung University, Tainan, Taiwan. Images were cropped and minimally processed, with brightness adjustments applied proportionally across all images using Microsoft PowerPoint (Microsoft, Redmond, WA, USA). Fluorescence intensity was quantified using ZEISS ZEN Blue software (Carl Zeiss AG, Oberkochen, Baden-Württemberg, Germany) with original unprocessed images acquired within the linear range of the detectors under consistent Zeiss confocal laser power settings; more detailed procedures can be found in [1].

**Quantitative RT-PCR**

RNA was isolated from 10 whole adult flies, or 30 adult fly heads, or 60 eye discs using TRIzol Reagent (Thermo Fisher Scientific, USA), following the manufacturer's protocol. The extracted RNA was then reverse-transcribed into cDNA using MScript RTase (GeneDirex, Taiwan). Quantitative real-time PCR (qRT-PCR) was performed with Power SYBR Green PCR Mix (Applied Biosystems, USA) and processed on a StepOnePlus PCR system. For normalization, *rpl32* was employed as the internal reference gene. The primers used in the analysis included:

*pepck1* F: GTGCCATCAACCCAGAGAAT

*pepck1* R: AGGTGTGTGGGCAGGTAAAG

*pepck2* F: TGACTGTCACGGACTGGTTG

*pepck2* R: TCGAGCCTCGTAAACCAAGG

*HexA* F: ATATCGGGCATGTATATGGG

*HexA* R: CAATTTCGCTCACATACTTGG

*Pfk* F: AGCTCACATTTCCAAACATCG

*Pfk* R: TTTGATCACCAGAATCACTGC

*Rpl32* F: AAGCTGTCGCACAAATGGCG

*Rpl32* R: GCACGTTGTGCACCAGGAAC

*G6Pase* F: GTCGATAGTGCACATTCTTTCGG

*G6Pase* R: GCCTTCCAAAACTCAAGCGTTC

*Tor* F: GAACAACCAGAGTCAGGCCA

*Tor* R: CTCCACAGAGCTTTCGTTGC

*4EBP1* F: CACTCCTGGAGGCACCAAAC

*4EBP1* R: GAGTTCCCCTCAGCAAGCAA

*S6* F: ACCGACAATATGAAGCTCAACG

*S6* R: CACAACCTGTCCCATACGCT

*GAPDH1* F: CCCAATGTCTCCGTTGTGGA

*GAPDH1* R: GTGTCGCTGAAGAAGTCGGT

*GlyP* F: TGGCTCATCTGTCCATCGT

*GlyP* R: ATACCGTTCGTCTTGTTCTGG

*TPS2* F: TGAAGATGCCATGAGAGTGCT

*TPS2* R: TTGCCGGTCAATGAAGGTTG

**RNA *in situ* hybridization**

Eye discs, dehydrated in 100% methanol, were transferred into nylon mesh baskets (designed for up to 50 embryos) and placed in 6-well plates at room temperature. The discs were rehydrated by moving the baskets sequentially through dilutions of methanol in 1x PBS. The discs were permeabilized with Proteinase K (10 µg/mL) at room temperature for 30 minutes. Proteinase K digestion was stopped by incubating the embryos in 4% paraformaldehyde (in 1x PBS, pH 7.5) for 20 minutes. The discs were washed four times in 1x PBST, for 5 minutes each, to remove residual paraformaldehyde. The tissues were transferred from the baskets to 1.5 ml sterile Eppendorf tubes. The embryos were prehybridized in 700 µl Hybridization Mix (HM) for 2-5 h at 70 ℃. The HM was replaced with 200 µl HM containing 30-50 ng of antisense DIG-labeled RNA probe, and hybridized overnight at 70 ℃. The discs were washed and incubated with anti-DIG antibody-alkaline phosphatase for overnight. The discs were then transferred back into HM with probe in small baskets, and washed in a 70 ℃ water bath with gentle agitation. The discs were gradually transitioned from HM to 2x SSC, which was then replaced with PBST. The discs were incubated in blocking buffer for 3-4 h at room temperature, followed by anti-DIG antibody solution diluted at 1/10,000 in blocking buffer overnight at 4 ℃, with gentle agitation. The discs were washed, and labeled larvae were stained for 4-11 h on Day 3. The larvae were then washed six times in PBST, at room temperature, with gentle agitation. The larvae were incubated three times in alkaline tris buffer with gentle agitation. The larvae were transferred from baskets to 1.5 ml microfuge tubes. The alkaline tris buffer was replaced with staining solution and incubated in the dark. The color reaction was monitored periodically under a dissecting scope and the reaction was stopped by transferring the larvae into 1.5 ml tubes filled with stop solution. The tissues were washed three times for 15 minutes, at room temperature, on a test tube rocker with gentle agitation. The stained tissues were stored in the dark in stop solution at 4 ℃ for several months. The primers used for *pepck1/2* in situ probe design were:

*pepck1* in situ F: GTGCCATCAACCCAGAGAAT

*pepck1* in situ R: AGGTGTGTGGGCAGGTAAAG

*pepck2* in situ F: CCAAAAGATTTCTGGGAGCA

*pepck2* in situ R: ATCGGCATTAATTGCTGGAC

**Glucose and trehalose assay**

Larval eye discs were dissected in PBS, and the material from 50 eye discs was homogenized in PBS containing 0.3% Triton X-100 and heated at 70 °C. The supernatant was collected after centrifugation at 14,000 rpm, for 10 minutes, in a cold room. Protein quantification was performed using Bradford Reagent, with 10 ml of supernatant incubated for 5 minutes. Trehalose levels in the entire body were assessed by treating 10 ml of supernatant with 0.2 ml of trehalase (Megazyme, E-TREH) at 37 °C for 30 minutes, following the manufacturer’s instructions for the glucose assay reagent (Megazyme, K-GLUC). After subtracting the quantity of free glucose, the resulting values were normalized to the protein levels in the supernatant.

**ChIP assay**

*Drosophila* (100 adults, sufficient for 2 independent immunoprecipitations) were homogenized in 5 ml of buffer A1 plus 1.8% formaldehyde (290 µl of 37% solution), at room temperature, using a Douncer with a type A pestle (10 strokes). After 15 minutes (total time from the start of homogenization), glycine solution was added to a final concentration of 225 mM (540 µl of 2.5 M solution for cross-linked mixture), mixed by vortexing, and incubated for 5 minutes on ice. The homogenate was transferred to a new 15 ml tube and centrifuged for 5 minutes, at 4000 g, at 4 ℃. The supernatant was discarded, 3 ml of buffer A1 was added, and the pellet was vortexed and spun down as done just prior. The washing step was repeated three times. The cross-linked material was then resuspended and incubated for 10 minutes, at 4 ℃, on a rotating wheel. The material was sonicated using a LINKO Ultrasonic Processor UP-300, on ice, with parameters set to power 20% (4 squares), time 4x30 seconds with 2-minute intervals. The sample was hybridized in a cold room overnight with rotation at 12 rpm. After overnight hybridization, the sample was incubated with 20 µl Dynabeads, at room temperature, for 2 h at 12 rpm. The beads were pelleted and the unbound fractions were collected (stored at -80 ℃). The beads were washed with 1 ml of washing buffer while rotating at 12 rpm. Immunocomplexes were eluted by adding 250 µl elution buffer (freshly prepared) and then rotated at room temperature for 15 minutes. The beads were pelleted and the eluate was collected. Crosslinks were reversed at 95 ℃ for 4 h with 200 mM NaCl (or at 65 ℃ overnight). Proteinase K digestion was performed at 57 ℃ for 1 h. DNA was recovered using a PCR clean kit (550 µl phenol was added and the sample was centrifuged at 14,000 g, for 15 minutes, at 4 ℃) with 20 µg glycogen as a carrier. Finally, input DNA was redissolved in 30 µl, and immunoprecipitated DNA was redissolved in 20 µl, autoclaved ddH_2_O.

**TUNEL assay**

Dissected larval eye discs were washed with PBS and fixed using 4% PFA, for 15 minutes, at room temperature. Subsequently, the eye discs were washed four times in PBS and then permeabilized by incubation in 100 mM sodium citrate and 0.1% Triton X-100, at 65 °C, for 30 minutes on a shaker set to 200 rpm. Next, the samples were washed twice with 3% BSA in PBS and then incubated in 1x TdT reaction buffer, at 37 °C, for 10 minutes, using the Click-iT™ Plus TUNEL Assay for In Situ Apoptosis Detection (Thermo Fisher Scientific, USA). After discarding the initial substrate, the tissues were incubated in the TdT reaction cocktail, at 37 °C, for 60 minutes, followed by two additional washes with 3% BSA in PBS. The tissues were then incubated with Click-iT™ Plus TUNEL Supermix, at 37 °C, for 30 minutes. Finally, the tissues were mounted using Vectashield dye (Vector Laboratories) and visualized under a Zeiss confocal microscope.

Reference:

1. Shihan, M.H., et al., *A simple method for quantitating confocal fluorescent images.* Biochem Biophys Rep, 2021. **25**: p. 100916.
